# Establishment of gastric signet ring cell carcinoma organoid for the therapeutic drug testing

**DOI:** 10.1038/s41420-021-00803-7

**Published:** 2022-01-10

**Authors:** Guoliang Li, Shuai Ma, Quanyou Wu, Defeng Kong, Zhenrong Yang, Zhaoru Gu, Lin Feng, Kaitai Zhang, Shujun Cheng, Yantao Tian, Wen Zhang

**Affiliations:** 1 State Key Laboratory of Molecular Oncology, Department of Etiology and Carcinogenesis, National Cancer Center/National Clinical Research Center for Cancer/Cancer Hospital, Chinese Academy of Medical Sciences and Peking Union Medical College, 100021 Beijing, China; 2 Department of Pancreatic and Gastric Surgery, National Cancer Center/National Clinical Research Center for Cancer/Cancer Hospital, Chinese Academy of Medical Sciences and Peking Union Medical College, 100021 Beijing, China; 3 Department of Immunology, National Cancer Center/National Clinical Research Center for Cancer/Cancer Hospital, Chinese Academy of Medical Sciences and Peking Union Medical College, 100021 Beijing, China

**Keywords:** Gastrointestinal cancer, Cancer models

## Abstract

Signet ring cell carcinoma (SRCC) has specific oncogenesis and phenotypic and treatment resistance heterogeneity. Systemic therapies are often ineffective, and predictive biomarkers to guide treatment are urgently needed. Tumor organoids have recently emerged as an ideal model for drug testing and screening. Here, we report gastric organoids established from tumor tissues comprising four SRCCs and eight non-SRCCs. Tumor organoids demonstrated different growth characteristics and morphologies. Changes in the original tumor genome were maintained during long-term culture from whole-exome sequencing (WES) analysis. Immunohistochemistry and H&E staining showed that the tissue characteristics of the primary tumor could be recapitulated. In addition, organoid lines successfully formed tumors in immunodeficient mice and maintained tumorigenic character. Different responses to 5-fluorouracil, oxaliplatin, docetaxel and irinotecan treatment were observed in SRCC and non-SRCC organoids. These results demonstrate that gastric organoid drug models, including SRCC, were highly similar to the original tumors in phenotypic and genotypic profiling and could be as living biomarkers for drug response testing.

## Introduction

Gastric cancer (GC) is one of the most common cancers and the third most common cause of cancer death globally [1, 2]. Although the incidence of GC has declined in recent decades, the incidence of the signet ring cell carcinoma (SRCC) subtype is increasing [3]. SRCC has specific oncogenesis, phenotypic and treatment resistance heterogeneity in GC. Compared to non-SRCC, SRCC is associated with a worse prognosis in advanced tumor stages [4–6]. It urgently needs convenient drug susceptibility test to clinical treatment strategy. Nowadays, two-dimensional (2D) cell culture and patient-derived xenografts (PDXs) were used for therapeutic drug screening. However, it is still difficult to accurately predict the sensitivity of individuals to therapy [7, 8]. Cancer cells have undergone substantial genetic changes and no longer retain the mutational profiles of the original tumors during 2D culture. 2D screening also cannot reflect the biological complexity and heterogeneity of the primary tumor [9, 10]. Although PDXs maintain the cancer mutational spectrum and 3D organization of the tumor, their generation is expensive, time-consuming, and resource-consuming [11]. Therefore, there is an urgent need for new drug screening or test models.

Recently, the successful establishment of patient-derived organoids (PDOs) for different tumors introduced new prospects for translational cancer research [12–15]. PDOs can maintain most of the primary tumor characteristics, such as self-renewal, multilineage differentiation, genetics, and histology. Once established, organoids can typically be cultured for a long time and can be expanded, cryopreserved, and genetically modified [16–18]. By testing chemotherapeutic drugs on patients’ tumors, it is possible to obtain the closest drug susceptibility data to the actual situation. Tumor organoids can be an individualized tumor model, which is expected to play an essential role in the individualized and precise treatment of tumors [16]. Several laboratories have established GC organoids from different histology and clinical stages as preclinical models for therapeutic drug screening [19–22]. However, the establishment of SRCC organoids is rarely reported.

In this study, we established 12 GC organoid lines from tumor tissues (four SRCC organoid lines and eight non-SRCC organoid lines). In addition, we provided a thorough phenotypic and molecular characterization of gastric organoid lines and their parental tumors, including the histological architecture, clinical marker expression, and genomic landscape. Then, we demonstrated the utility of gastric organoid lines as a preclinical model for drug testing to identify new therapeutic targets and potential applications to advance personalized medicine.

## Result

### Human gastric cancer organoids are established from cancer tissues

**Fig. 1:**
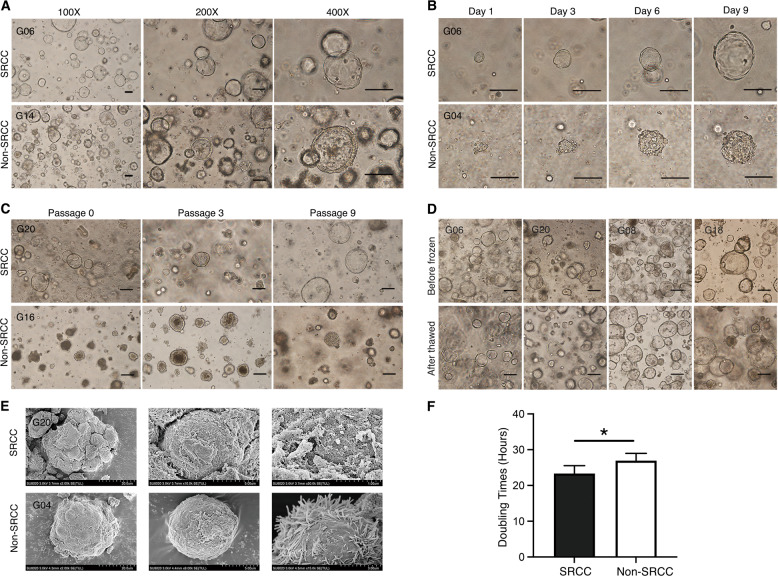
Bright field performance and growth characteristics of GC organoids. **A** The growth of gastric SRCC (G06) and non-SRCC (G14) organoids under different magnifications. Scale bars, 100 μm. **B** The growth process of SRCC (G06) and non-SRCC (G04) organoids. Scale bars, 100 μm. **C** The characteristics of long-term culture of SRCC (G20) and non-SRCC (G16) organoids. Scale bars, 100 μm. **D** Bright field appearance of SRCC (G06, G20) and non-SRCC (G08, G18) organoids before cryopreservation and after resuscitation. **E** The appearance of SRCC (G20) and non-SRCC (G04) organoids under the electron microscope. **F** Comparison of cell doubling times, four SRCCs and eight non-SRCC organoid lines. Data are presented as the mean ± SD. *p < 0.05.

Using 3D organoid culture technology, 12 cases of GC organoid lines form 26 collected GC tissue samples were successfully established, including four cases of SRCC and eight cases of non-SRCC. The total organoid establishment efficiency was 46% (12/26), SRCC was 50% (4/8), and non-SRCC was 44% (8/18). The detailed clinicopathological information is shown in Tables 1 and 2. The success rate of organoid culture has no relationship with clinical characteristics. Under optimized conditions, 3D organoids generated round shapes within several days. As shown in Fig. 1A, organoids had very different morphological characteristics from traditional 2D cultures. To evaluate the growth process of organoids, we performed continuous observation research (Fig. 1B). The long diameter of a single organoid can reach ~100 μm after one week of culturing. In addition, we recorded the growth status of organoids every 3 h for 72 h. Supplementary Video 1 shows the growth status of G06 (SRCC), and Supplementary Video 2 shows the growth status of G04 (non-SRCC). Gastric organoids displayed a glandular, solid, cystic, grape-like, or mixed morphology discohesive growth pattern to varying degrees that mimicked the in vivo tumor, albeit with some differences for individual cases, potentially attributed to regional heterogeneity (Fig. 1A, C, D). Regardless of whether it is SRCC or non-SRCC, once the organoids are successfully established, they can be cultured for a long time. At present, G20 (SRCC) and G16 (non-SRCC) have been cultured for 6 months, and the morphological characteristics of the organoids have not followed the increased number of changed passages (Fig. 1B). Each of the 12 organoid lines that we successfully cultivated was cryopreserved and resuscitated. As shown in Fig. 1D, the cell morphology and characteristics of the organoids before and after cryopreservation did not significantly change. By scanning electron microscopy, as shown in Fig. 1F, GC organoids are spherical structures composed of many tightly connected cells. Then, the 12 organoid lines were divided into two groups: SRCC group and non-SRCC group. The cell proliferation test shows that the cell doubling time of SRCC was 23.33 ± 2.22 h, and the cell doubling time of non-SRCC was 26.93 ± 2.04 h (*p* < 0.05, Fig. 1E). Our data show that SRCC has a higher organoid proliferation rate than non-SRCC, and there is no difference between SRCC organoids and non-SRCC organoids from a morphological viewpoint.

**Table 1:** Baseline characteristics of the patients.

Characteristics	Total (*N* = 26)	Organoids culture		*p*
		Success (12)	Fail (14)	
Age (years)				
≥60	13	4	9	*p* = 0.24
<60	13	8	5	
Sex				*p* = 0.68
Male	18	9	9	
Female	8	3	5	
Differentiation				*p* = 0.27
Middle–High	13	4	8	
Low	13	8	6	
Subtype				*P* > 0.99
SRCC	8	4	4	
Non-SRCC	18	8	10	
TNM stage				*p* = 0.17
I–II	6	1	5	
III–IV	20	11	9

**Table 2:** Clinical data of gastric cancer patient-derived organoids.

Patient ID	Sex	Age	Diagnosis	TNM (UICC stage)	Tumor differentiation	Histology (Lauren classification)	Tumor site
G01	M	56	Adenocarcinoma	T4aN3bM0 (IIIC)	Poor	Diffuse	Antrum
G04	F	70	Adenocarcinoma	T4aN3bM0 (IIIC)	Poor	Mixed	Cardia
G06^a^	F	55	Adenocarcinoma	T1aN0M0 (IA)	Poor	Diffuse	Body
G08	F	66	Adenocarcinoma	T2aN3bM1(IV)	Moderate	Mixed	Body
G10	F	69	Adenocarcinoma	T4N3aM0 (IIIA)	Poor	Intestinal	Body
G14	F	56	Adenocarcinoma	T4N3aM0 (IIIA)	High	Intestinal	Cardia
G16	F	69	Adenocarcinoma	T4aN3aM0 (IIIB)	Moderate	Mixed	Cardia
G18	F	56	Adenocarcinoma	T4aN3aM0 (IIIB)	Moderate	Intestinal	Antrum
G20^a^	F	48	Adenocarcinoma	T4aN3aM0 (IIIB)	Poor	Diffuse	Body
G22^a^	M	58	Adenocarcinoma	T4aN2M0(IIIA)	Poor	Diffuse	Body
G23	M	47	Adenocarcinoma	T4aN3aM0 (IIIB)	Poor	Diffuse	Body
G25^a^	F	55	Adenocarcinoma	T3NM0(IIA)	Poor	Diffuse	Cardia

^a^The patients (G06, G20, G22, and G25) were diagnosed of SRCC.

### Gastric cancer organoids retain the genetic characteristics of cancer tissues

**Fig. 2:**
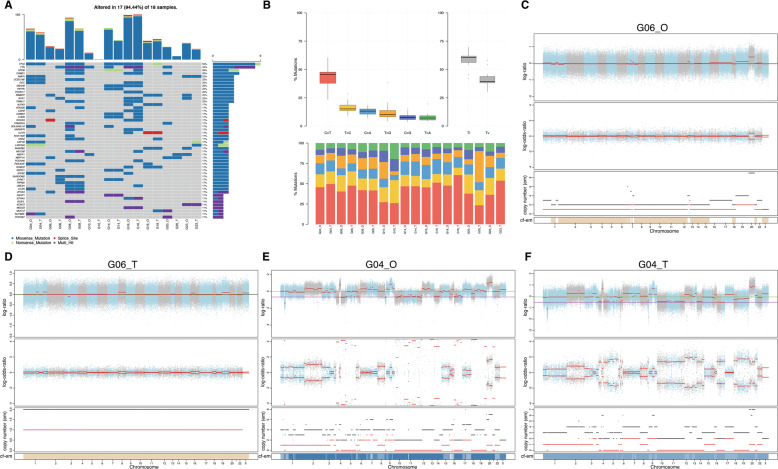
GC organoids can maintain the genomic characteristics of the original tissue. **A** The top 50 mutant genes are obtained by analyzing the SNV of organoids and matched primary tissue. The legend showed the types of mutations. **B** The mutation types and proportions of all samples. **C**–**F** Use FACETS software to analyze CNV of G06_O, G06_T, G04_O, and G04_T. O organoids, T original tissue.

To determine whether the successfully cultured GC organoid line maintains the genetic mutations of the original tumor tissue, whole-exome sequencing (WES) analysis was performed for 9 organoids and matched tissues. SNV and CNV analyses show that organoids obtained by culture have substantial similarity with the primary tumor (Fig. 2). First, we used muTect to analyze the somatic cell SNV of the paired sample and annotated it with ANNOVAR to screen the mutation sites and count the mutation frequencies of the corresponding genes; the basic information of SNV is shown in Supplementary Fig. 1. Then, we selected the top 50 genes with the highest mutation frequency to make an oncoplot (Fig. 2A). The types of mutations in GC organoids and tumor tissue samples include missense mutations, splice sites, nonsense mutations and multiple hits, and common GC mutated genes such as TP53, TTN, and CSMD1 are shown in both of them. In addition, it is not difficult to visit the oncoplot above that regardless of whether it is in SRCC or non-SRCC, organoids and paired primary lesions have similar SNV mutation characteristics. The base substitutions of all samples included C > T, T > C, C > A, T > G, C > G, and T > A; among them, C > T had the highest substitution frequency GC (Fig. 2B). Further analysis shows that in matched organoids and tissue samples, the proportions of different types of base substitutions also had remarkable similarities. Figure 2C, D shows the CNV of SRCC (G06) organoids and matched tumor tissues. In the case of mutation, the top and middle diagrams show the distribution of logR and logOR values of all mutation sites in the chromosome. The bottom diagram is the copy number of the sample obtained by the CBS algorithm. Similar Fig. 2E, F show the CNV mutations of non-SRCC (G04) organoids and matched tumor tissues. The CNV analyze of other organods and paired tissues was shown in Supplementary Fig. 2. Comparing the variation of CNV, we suggested that the organoids obtained by 3D culture retain the characteristics of CNV variation of the original tissue in the genome of chromosome breakpoints, chromosome fragmentation, and chromosome ploidy changes.

### Gastric cancer organoids maintain the histology of original cancer tissues

**Fig. 3:**
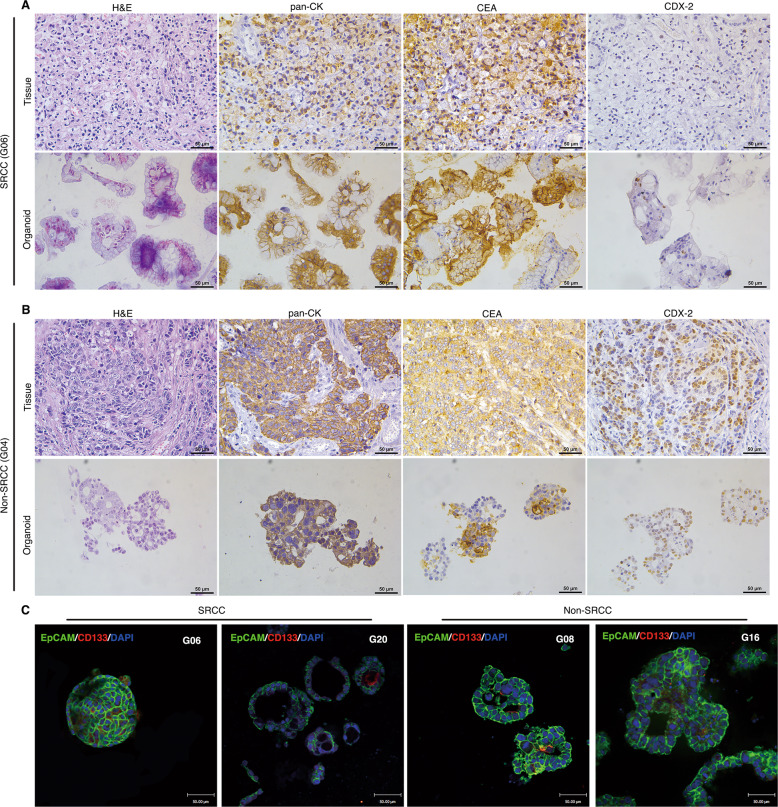
Gastric cancer organoids can reproduce the histological characteristics of the primary tissue. **A** H&E and IHC staining (pan-CK, CEA, and CDX-2) showed that the successfully cultured SRCC (G06) organoids and primary tumors are consistent in histological characteristics. Scale bars, 50 μm. **B** H&E and IHC staining (pan-CK, CEA, and CDX-2) showed that non-SRCC (G04) organoids were consistent with the primary tumor but different from SRCC, Scale bars, 50 μm. **C** Immunofluorescence shows that SRCC (G06 and G20) and non-SRCC (G08 and G16) have similar characteristics in the expression of EpCAM (Green) and CD133 (Red). Scale bars, 50 μm.

Further, we conducted H&E and IHC analyses on the paired organoids and tissues. SRCC is a specific type of GC where the nucleus is squeezed to one side of the cell due to mucus inside the cell. As shown in Fig. 3A, SRCC organoids (G06) can reproduce the characteristics of the primary tissue on H&E staining. Simultaneously, IHC was used to analyze the typical GC marker expression of pan-CK, CEA, and CDX-2. The results showed that both pan-CK and CEA, but not CDX-2, were expressed in G06 primary tumors and organoids. Moreover, we performed the same H&E and IHC analyses on non-SRCC G04 (Fig. 3B). H&E staining showed irregular glandular ducts or acini in G04 organoids, which are very similar to the shape of the primary tumor. Interestingly, the tumor-related markers pan-CK, CEA, and CDX-2 were widely expressed in non-SRCC G04 organoids and primary tissues. H&E and IHC staining of other organoids and paired primary tissues were shown in Supplementary Figs. 3 and 4. The data shown that pan-CK and CEA were expressed in all organods and paired tissues, while CDX-2 was expressed differently, but no difference between non-SRCC and SRCC. To further understand the morphological and histological characteristics of GC organoids, we performed an immunofluorescence analysis on the tumor epithelial adhesion molecule EpCAM and the tumor stem cell marker CD133 (Fig. 3C). The results showed that almost all cells expressed EpCAM in both SRCC and non-SRCC organoids. Some cells in the organoid expressed the stem cell marker CD133, which may be related to the self-renewal ability of the organoid and the maintenance of the spatial structure. The expression of CD133 in organoids and their tissues were displayed in Supplementary Fig. 5. CD133 was also detected in organoid and paired tissues by IHC staining. Thus, GC organoids obtained by 3D culture in vitro can reproduce the morphology and histological characteristics of the primary tumor.

**Fig. 4:**
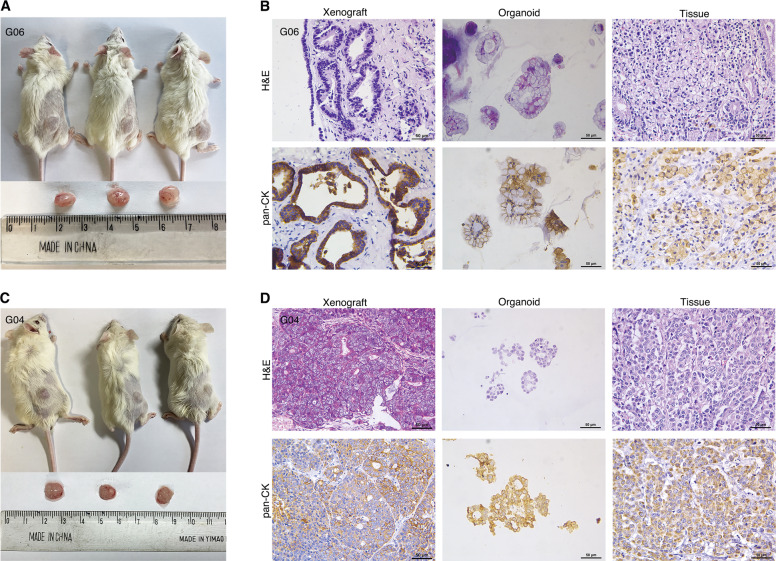
Tumorigenesis of the GC organoids in vivo. **A**, **C** The appearance of the SRCC (G06) and non-SRCC (G04)organoid tumors in immunodeficient mice. **B**, **D** H&E and IHC (pan-CK) staining of xenograft tumor, organoids, and primary tissue about G06 and G04. Scale bars, 50 μm.

### Tumorigenicity analysis of gastric cancer organoids in immunodeficient mice

To evaluate the tumorigenicity of GC organoids, we transplanted organoids into immunodeficient mice and observed their growth status. As shown in Fig. 4A–C, four organoid lines (SRCC: G06 and G20, Non-SRCC: G04 and G14) were used for mouse subcutaneously. After ~45 days, the G06 and G04 organoid lines successfully formed tumors under the skin of the mice. The H&E staining results showed that xenografts of the SRCC organoid line (G06) were slightly different from those of the 3D organoid culture (Fig. 4B). This difference may be due to complex reasons in vivo involving a change in the structure of the signet ring cells. The phenomenon that the nucleus shifted to one side was not obvious, but a glandular tube-like structure was formed in the xenograft tumor, which is consistent with the general pathological characteristics of GC. In addition, the IHC results showed that pan-CK was widely expressed in transplanted tumors, which is consistent with the organoids and primary tissues (Fig. 4B). Tumorigenesis and H&E and IHC analyses of non-SRCC organoid line (G04) xenografts are shown in Fig. 4C, D. The difference between non-SRCC and SRCC in vivo was due to the proportion of the extracellular matrix composition.

### Gastric cancer organoids for patient-specific drug trials in vitro

**Fig. 5:**
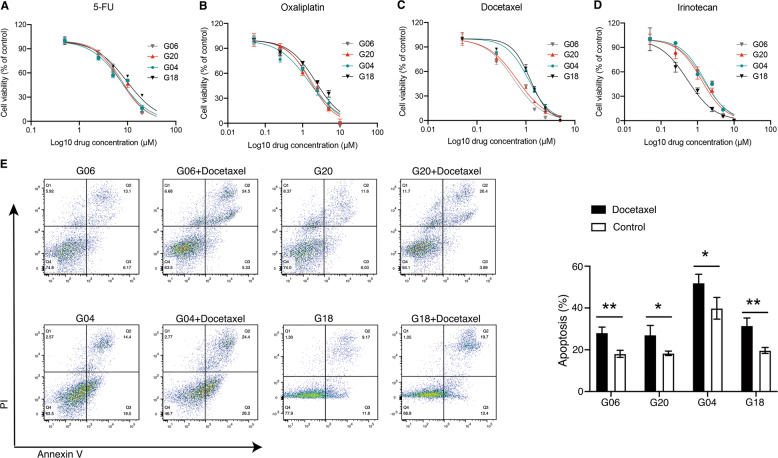
Different treatment responses of GC organoids to conventional chemotherapeutics. **A**–**D** The drug dose-response curve of SRCC (G06 and G20) and non-SRCC (G04 and G18) after treated with 5-FU, oxaliplatin, docetaxel, and irinotecan; each data point represents three biological replicates, with error bars representing mean ± SD. **E** Apoptosis assay using annexin V/PI flow cytometry (one example of three independent experiments is shown). Data are presented as the mean ± SD. *p < 0.05, **p < 0.01.

Here, four organoid lines (SRCC: G06 and G20, non-SRCC: G04 and G18) were used to preliminarily assess for drug sensitivity testing in vitro. According to the NCCN guidelines, four commonly used GC clinical drugs were used for drug screening: 5-FU, oxaliplatin, docetaxel, and irinotecan. Six different concentrations of each drug were evaluated to test drug sensitivity. As shown in Fig. 5A–D, the IC50 values of G06, G20, G04 and G18 for 5-FU were: 7.68 ± 0.97, 7.23 ± 0.82, 7.212 ± 1.04, 9.55 ± 1.51 μM; those for oxaliplatin were: 1.74 ± 0.34, 1.63 ± 0.25, 1.47 ± 0.33, 2.23 ± 0.48 μM; those for docetaxel were: 0.57 ± 0.11, 0.66 ± 0.09, 1.13 ± 0.15, 1.23 ± 0.1 μM; and those for irinotecan were: 1.45 ± 0.35, 1.28 ± 0.24, 1.55 ± 0.25, 0.53 ± 0.1 μM, respectively. There was no significant difference in IC50 between 5-FU and oxaliplatin in these four organoid lines, which is consistent with some previous clinical studies [23, 24]. Compared with non-SRCC organoid lines, the IC50 value of SRCC organoid lines (G06 and G20) against docetaxel was lower. Among the other three drugs, there was no significant difference between the two types of organoids, which suggests that SRCC was more sensitive to docetaxel. The divergent response to docetaxel treatment was further analyzed by annexin V/PI showing the difference in their response (Fig. 5E). In G06 and G20 organoids, annexin V staining increased from on average 18.1–28.1% and 18.3–27.1% for apoptotic cells upon treatment with 1.5 µM docetaxel. On the other hand, in G04 and G18 organoids, annexin V staining increased from on average 39.8–52.0% and 19.7–31.5% for apoptotic cells upon treatment. Overall, both SRCC and non-SRCC organoid lines exhibited heterogeneous responses in drug sensitivity screen assays.

## Discussion

Our study demonstrated the possibility of establishing organoid biobanks of GC, including four SRCC lines and eight non-SRCC lines from GC tissue. Among them, the failure of culture in six cases may be due to tissue contamination, which is related to the collection of materials, and the failure of passage in the other eight cases, which may be related to the pathological characteristics of the patients or limitations of the culture technology. In our study, comprehensive characterization of GC organoid lines confirmed that they recapitulated the features of the corresponding parental tumors in terms of histological architecture, cancer driver gene mutations, CNVs, and SNVs after culture in vitro.

Since GC may have no obvious clinical symptoms even in the advanced stage, many patients are diagnosed in unresectable stages, which is associated with poor prognosis [1, 25]. Currently, chemotherapy is the most common treatment for advanced-stage GC. However, it is not clear whether a specific chemotherapy regimen is justified. Although identification of targetable genomic alterations by high-throughput sequencing is paramount for precision cancer care of patients with advanced disease, only a tiny proportion of patients benefit from WES-guided drug screens. There are no reliable biomarkers to predict its efficacy, which may be influenced by histological classification, grading and staging. Traditional methods such as PDXs and traditional cancer cell lines have limitations in predicting responses to drug therapies. Advantages of organoid cultures are the short time frame to establishment, low cost and ease of manipulation compared with PDXs. Although drug screens on large 2D cancer cell line collections have provided major insights into drug response, their substantial genetic changes may have contributed to the high failure rate of screening drugs in clinical trials [9]. Tumor organoids can better reproduce the genetic and histological characteristics of the primary tumor. Compared with traditional methods, organoids may be an excellent model to identify and test anticancer drugs.

Organoids have proven to be a useful biomarker tool to guide and tailor treatment [26–28]. For establishment, it is considered successful that organoid lines can be expanded for at least five passages. Organoid treatment response tests were completed in less than four weeks, which indicates that this model can produce treatment recommendations within a clinically meaningful time frame. Previous studies have reported the use of GC organoids to test drug responses [19, 21, 22]. However, to our knowledge, there are few reports on the establishment of SRCC organoids. SRCC cells are a challenging type of organoid to establish because they are closely related to stromal cells, and the rapid growth of mesenchymal cells makes it difficult to separate from fibroblasts. Moreover, signet ring cells have a relatively long quiescent period before rapid proliferation, and improper culture can lead to cell death [29].

Although SRCC is considered less chemically sensitive than non-SRCC, whether it is insensitive to all current chemotherapy regimens remains inconclusive. Messager et al. [30] found that chemotherapy with epirubicin-cisplatinum-5-fluorouracil provided no survival benefit in patients with SRCC. Chen et al. [31] discovered a benefit of docetaxel-based chemotherapy in mixed SRCC compared with oxaliplatin-based chemotherapy. Pernot et al. [32] reported that chemotherapy with docetaxel-5-FU-oxaliplatin (TEFOX) appeared to be an effective treatment in advanced gastric SRCC and had an acceptable safety profile. These data suggest that the curative effect of SRCC could be improved by using intensive treatment with taxane-based chemotherapy. In this study, we found that organoid lines varied in their responses to drugs, and SRCC might be highly sensitive to docetaxel. This result was similar to the conclusions about SRCC in the above studies. Clinical trials (NCT01249859 and NCT01717924) are studying the SRCC drug sensitivity, but those trials lack preclinical patient drug model tests. Our experimental protocol for SRCC can be used as a part of future clinical trials for drug screening. In addition, it shown that GC organoids can generate xenografts in immunodeficient mice, which enables in vivo drug testing. Since GC is a particularly heterogeneous disease, our organoid biobank can complement previous studies and increase the availability of in vitro drug models.

The most significant limitation of the GC organoid model is the lack of a tumor microenvironment such as stromal cells and immune cells. Thus, future studies that involve co-culturing these GC organoids with patient immune cells will serve as an excellent model to study the viral gene regulation and may uncover methods to reactivate genes for immune recognition as a therapeutic strategy [33, 34]. Second, the success rate in our organoid culture was approximately 50%. The success rate difference may be due to severe necrosis that usually affects gastric tumor tissues. And to increase the chance of optimal organoid formation, an appropriate increase in the concentration of Y-27632 in the first two generations of medium may be a solution. Third, this study was hampered by the small sample size of patients. It is necessary to further expand the sample size and verified.

In conclusion, organoids provide an opportunity to bridge the gap between traditional cancer cell lines and PDXs. Gastric-containing SRCC organoid-based high-throughput drug testing can generate a link among GC, genetics and clinical trials to design personalized therapies.

## Materials and methods

### Patients and sample collection

From August 2020 to January 2021, a total of 26 gastric tissues were obtained from patients who underwent surgery at Cancer Hospital, Chinese Academy of Medical Sciences and Peking Union Medical College. Baseline characteristics of the patients was described in Table 1. The detailed clinicopathological information of GC patient-derived organoids is shown in Table 2. The patients (G06, G20, G22, and G25) were diagnosed of SRCC. This study was approved by the ethics committee of the Cancer Institute and Hospital of the Chinese Academy of Medical Sciences (No.14-067/857). Written informed consent was obtained from the enrolled patients.

### Organoid cultures

GC tissue specimens were processed by anatomical shredding, collagen digestion, lysing red blood cells, etc. Then the tumor cells were embedded in the Matrigel growth factor reduced basement membrane matrix(corning, #356321, USA) to form organoids. The fresh medium was changed every 3 days and organoids were passaged every 2 weeks. Details are provided in Supplementary Materials and Methods.

### Genomic analysis

DNA was extracted from gastric tumor tissues with matched normal tissues and matched gastric organoids using the DNeasy Blood & Tissue Kit (Qiagen, Germany) according to the standard protocol. Samtools mpileup and bcftools were used to do the variant calling and identify SNP, InDels. The somatic SNV was detected by muTect, the somatic InDel by Strelka. Then we use the maftool R package to visualize the results [35]. And copy number was analyzed by FACETS (version 0.6.1), using default parameters [36].

### H&E, immunohistochemistry, and immunofluorescence

Gastric tissues and organoids were fixed in 4% paraformaldehyde followed by a standard staining protocol concerning dehydration, paraffin embedding, sectioning and H&E staining. Immunohistochemistry of organoids and tissue sections for pan-CK, CEA, and CDX-2 was performed according to standard procedures. For immunofluorescence staining, the samples were incubated with primary antibodies, including EpCAM and CD133. Then, primary antibodies were detected by incubating with Alexa Fluor 488 and Alexa Fluor 568. Further information is given in Supplementary Materials and Methods.

### Establishment of organoid-derived xenografts

Gastric organoids were separated from Matrigel by TrypLE Express for single cells followed by incubation for 5–10 min. One hundred microliters of dissociated organoids were mixed in Matrigel and subcutaneously injected into NPI mice (NOD-*Prkdc*^em1IDMO^-*Il2rg*^em2IDMO^, 4–6 weeks, Beijing IDMO CO., Ltd). Monitor tumor growth by vernier calipers of the injection site twice per week. Then, the immunodeficient mice were euthanized when the tumor reached 1000 mm^3^, and the tumors were obtained for morphological study. Mice experiments were approved by the Experimental Animal Ethical Committee of Cancer Hospital, Chinese Academy of Medical Sciences and Peking Union Medical College (NCC2020A304). The mice experiment was carried out at Cancer Hospital, Chinese Academy of Medical Sciences and Peking Union Medical College from December 2020 to May 2021. All mice studies were performed according to National Institutes of Health guidelines for the care and use of laboratory animals. Mice were anesthetized with isoflurane gas and sacrificed by cervical dislocation. Mice carcasses were collected and incinerated by a licensed contractor (Beijing Solid Waste Logistics CO., LTD).

### Drug tests assays

Three days post-seeding, media was removed and replaced by media containing six concentrations of 5-FU, oxaliplatin, irinotecan, and docetaxel. After 72 h, the maximum half-inhibitory concentration (IC50) was determined to test drug sensitivity. The divergent response to docetaxel treatment was further analyzed by annexin V/propidium iodide flow cytometry. The detailed procedures are provided in Supplementary Materials and Methods.

### Statistical analysis

Statistical analyses were performed using Student’s *t* test and compared SRCC versus non-SRCC patients with *p* values indicated in the table or in the figures. IC50 calculation was determined with non-linear regression using Prism (GraphPad Prism 9). Results were expressed as mean ± standard deviation.

## Supplementary information

Graphical abstract

Supplementary Video 1

Supplementary Video 2

Supplementary Information

Supplementary Materials and Methods

Supplement Figure 1

Supplement Figure 2

Supplement Figure 3

Supplement Figure 4

Supplement Figure 5

Supplement legends

Highlight

source_code

Author Contributions Section

Declaration of Interest Statement

Informed consent form

## Data Availability

The datasets used and/or analyzed during the current study are available from the corresponding author on reasonable request.
