# Supplementary figures and images for: Establishment of gastric signet ring cell carcinoma organoid for the therapeutic drug testing
Source: Cell Death Discov. 2022 Jan 10;8:6. doi: 10.1038/s41420-021-00803-7 (PMC8748936; doi:10.1038/s41420-021-00803-7)

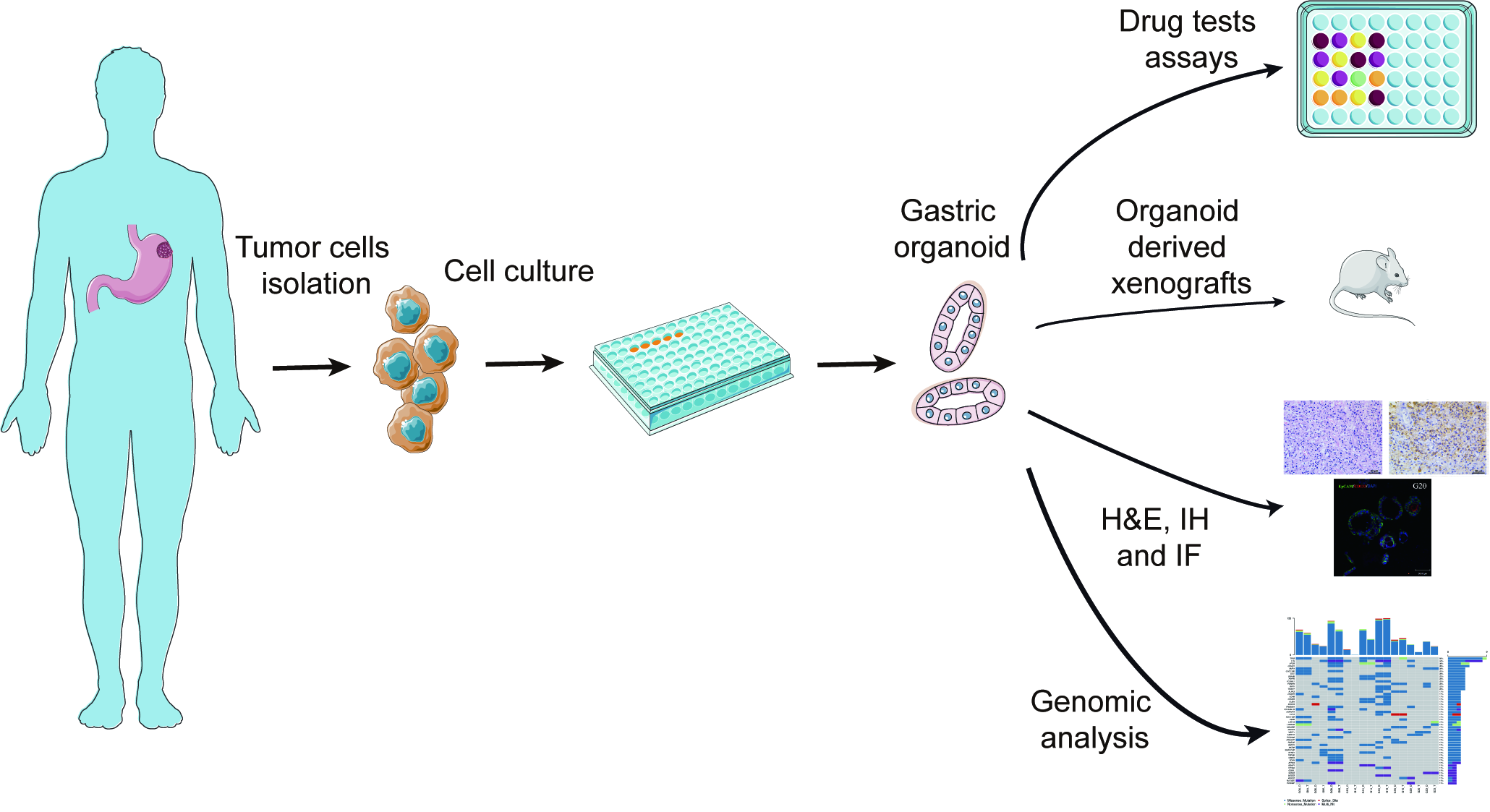

Supplement: Supplementary file 1 — Graphical abstract [file 41420_2021_803_MOESM1_ESM.tif]

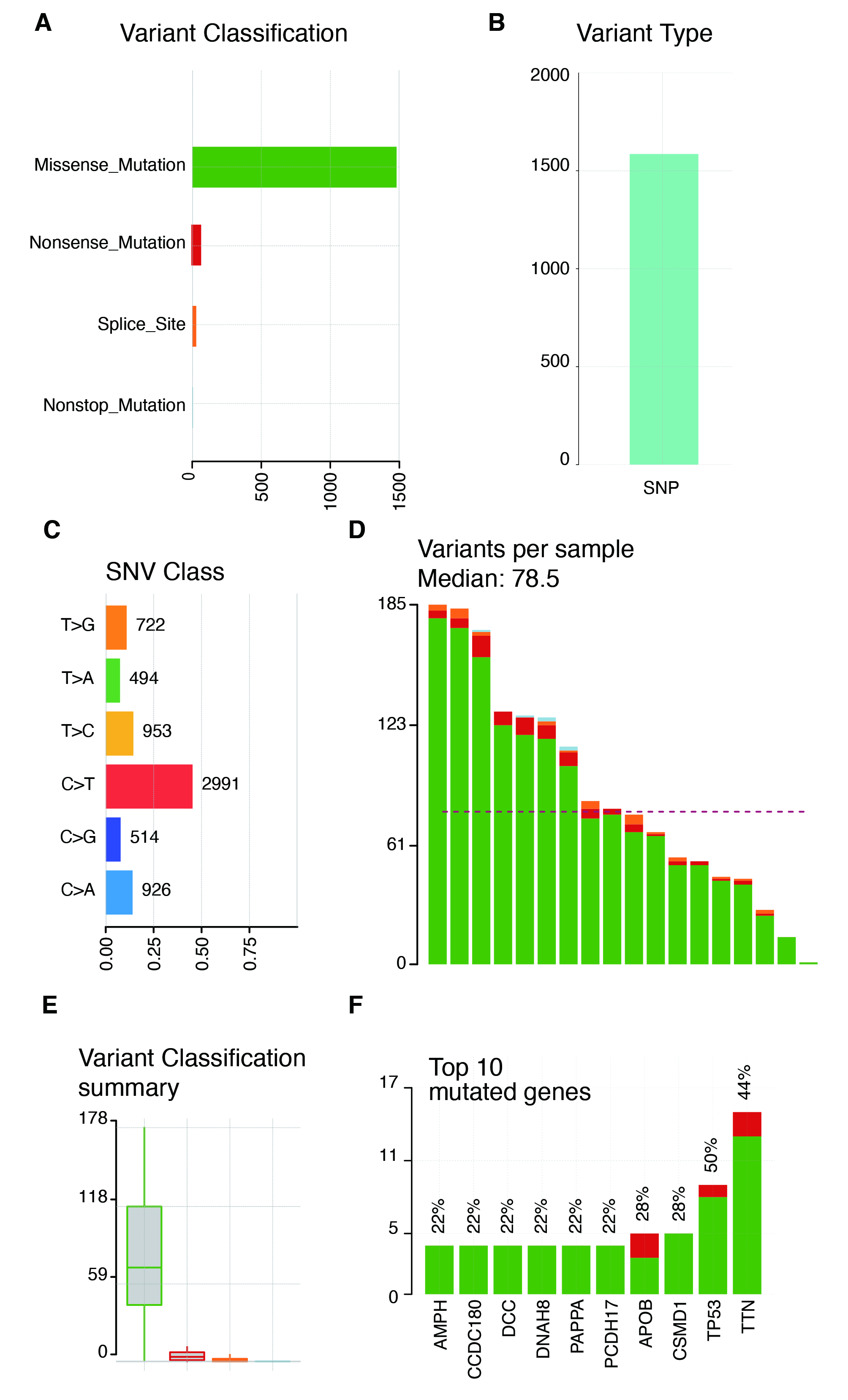

Supplement: Supplementary file 6 — Supplement Figure 1 [file 41420_2021_803_MOESM6_ESM.tif]

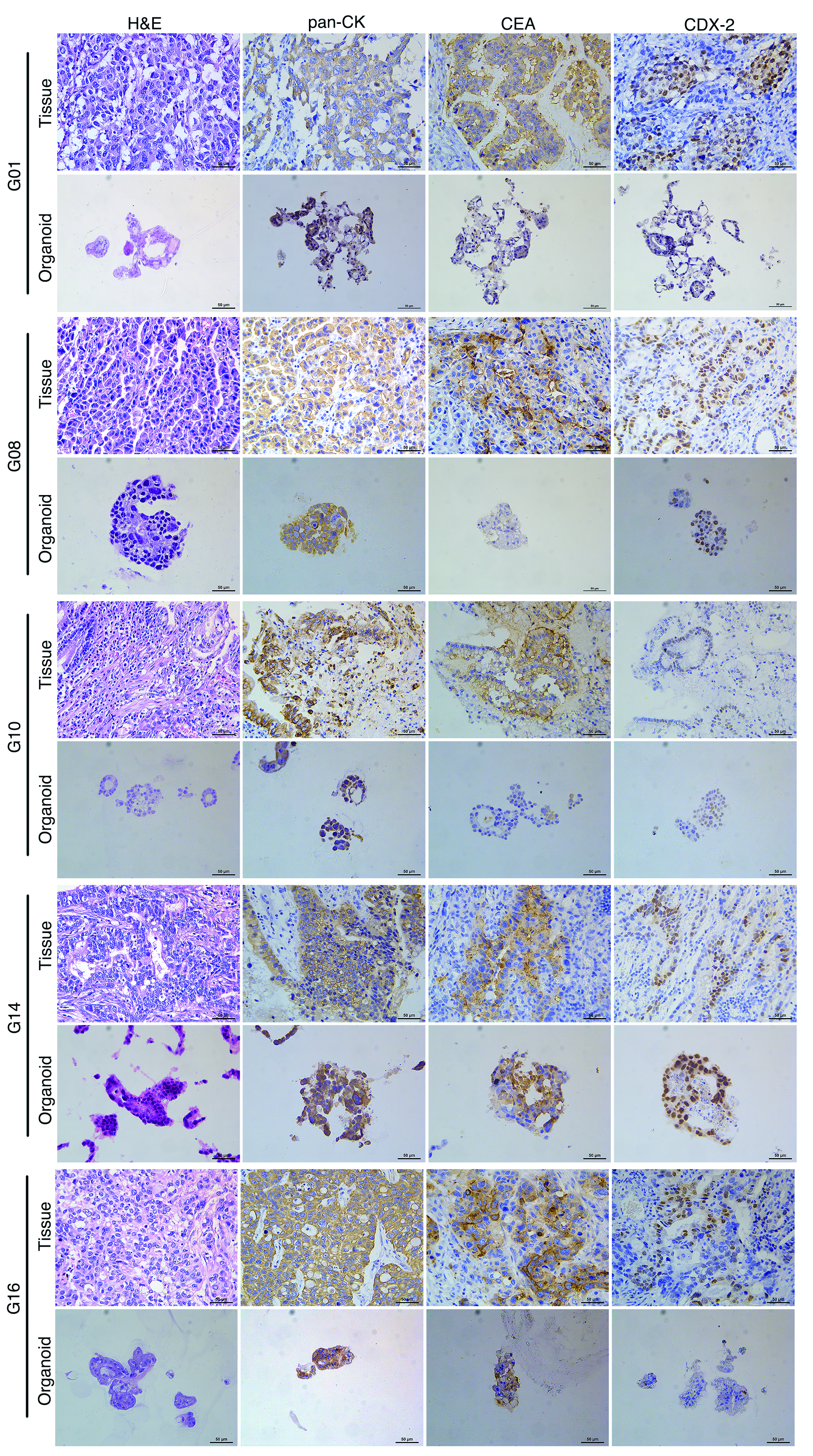

Supplement: Supplementary file 8 — Supplement Figure 3 [file 41420_2021_803_MOESM8_ESM.tif]

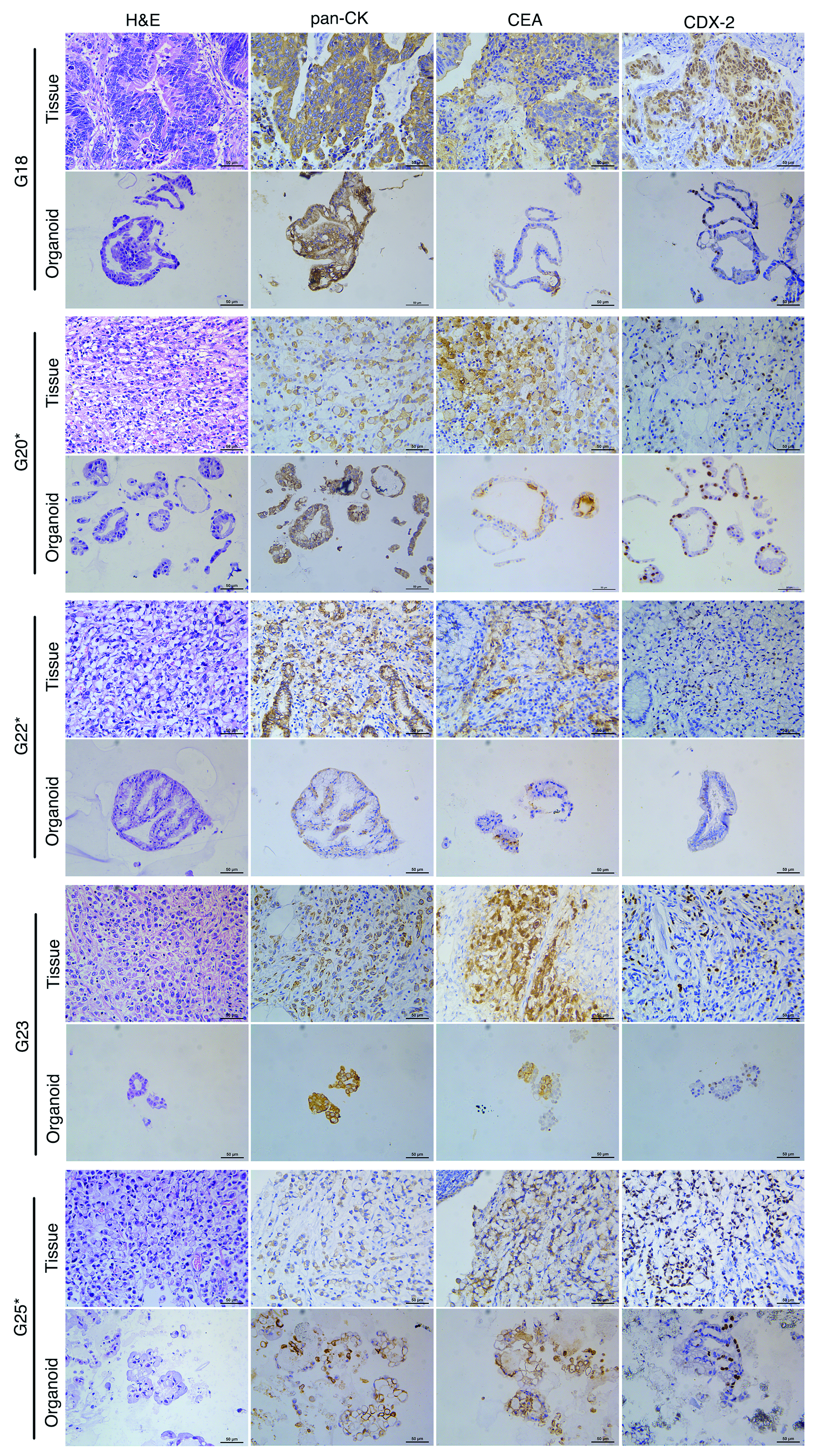

Supplement: Supplementary file 9 — Supplement Figure 4 [file 41420_2021_803_MOESM9_ESM.tif]

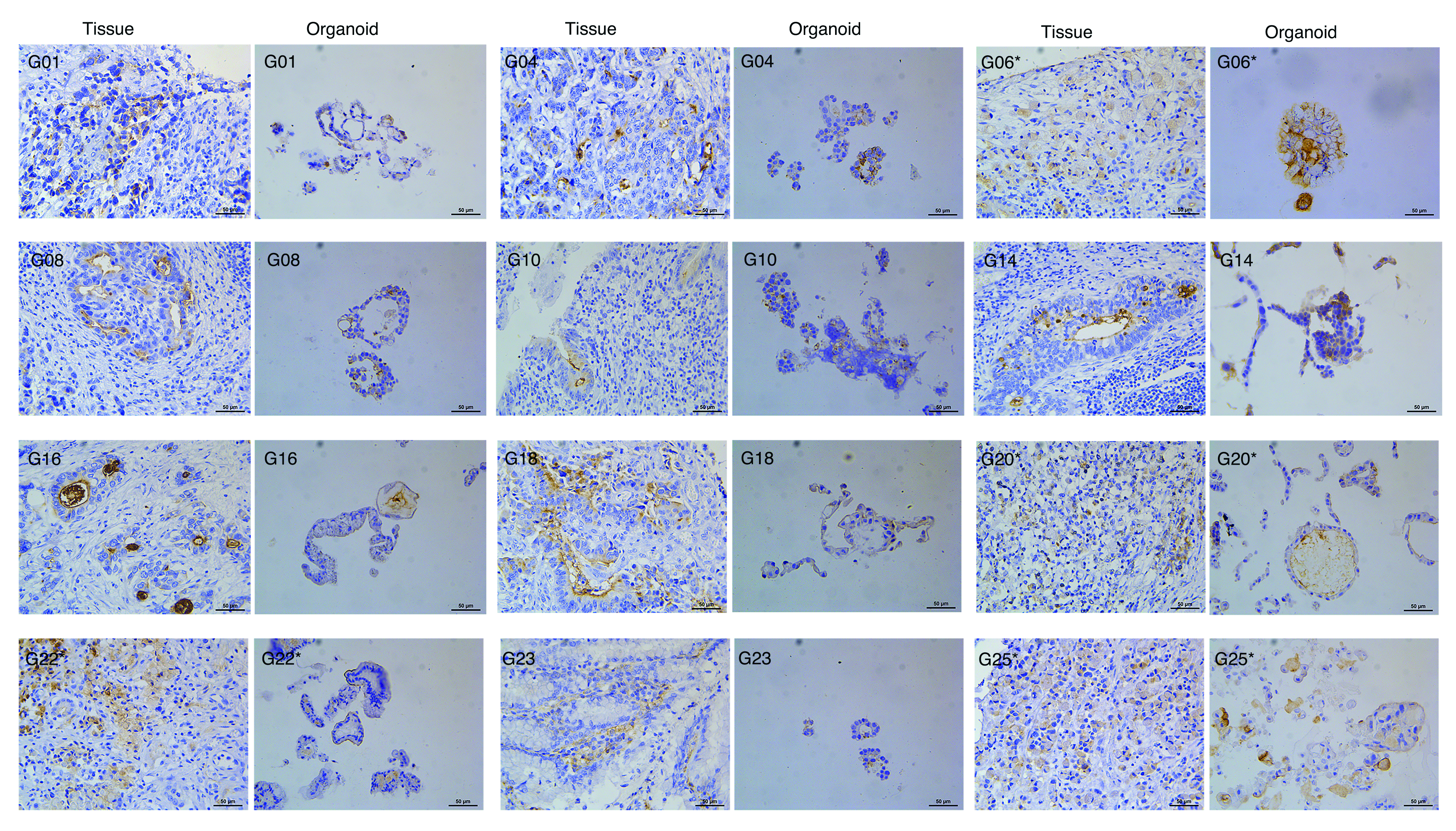

Supplement: Supplementary file 10 — Supplement Figure 5 [file 41420_2021_803_MOESM10_ESM.tif]
