# Supplementary material for: Establishment of gastric signet ring cell carcinoma organoid for the therapeutic drug testing
Source: Cell Death Discov. 2022 Jan 10;8:6. doi: 10.1038/s41420-021-00803-7 (PMC8748936; doi:10.1038/s41420-021-00803-7)
Supplement: Supplementary file 4 — Supplementary Information [file 41420_2021_803_MOESM4_ESM.pdf]

Supplementary Materials and Methods

1. Tumor cells isolation and establishment of human gastric organoid cultures

Gastric cancer tissues were washed in the cold PBS with 1X penicillin/streptomycin (P/S) three times. Use scalpels and scissors to mince human gastric tissues into small pieces (1–5 mm^3) in a 10-cm culture dish. Then, tissue was inserted into a gentleMACS C Tube with a volume of 5 ml digestion buffer (advanced DMEM/F12 containing 1X P/S, 2.5% FBS, 0.6mg/mL collagenases, 20mg/mL hyaluronidase and 10mM Y-27632). The C tube was then placed onto the gentleMACS Octo dissociator with heater. After tumor tissue dissociation, tissue debris were removed by passing the mixture through a 70uM cell strainer before centrifugation at 300g for 5 min at 4 °C. The pellets were resuspended in 10 ml adDMEM/F12 +/+/+ (adDMEM/F12 containing penicillin/streptomycin, 10 mM HEPES and 2 mM GlutaMAX) and centrifuged again at 300g for 5 min at 4 °C. If there were red blood cells precipitation, add 1-2 ml red blood cell lysis buffer to lyse at room temperature for 3 minutes, and add 10 ml AdDMEM/F12 +/+/+ before centrifugation at 300g for 5 min at 4 °C， then cells were counted with the Cellometer ® Auto 2000 and ViaStainTM AOPI Staining Solution (NexcelomBioscience, USA), and appropriate cell dilutions were done in cold Matrigel growth factor reduced basement membrane matrix(corning, 356321). 40ul drops (~20000 cells) were plated in the middle of one well of a pre-warmed 24-well plate at 37°C for 10-15 mins. After the Matrigel solidified, 400 ul of gastric organoid medium (advanced DMEM/ F12, 10 mM HEPES and 2 mM GlutaMAX, 100U/ml P/S, 1X B27 supplement, 1.25mM N-Acetylcysteine, 5mM Nicotinamide, 100ng/ml Noggin, 100ng/ml Wnt3a, 500ng/ml R-Spondin 1, 50ng/ml EGF, 25ng/ml EGF2, 200ng/ml FGF10, 2mM A83-01, 10mM Y-27632, 1nM Gastrin) was added to each well and plates transferred to the CO2 incubator (5% CO2, 37°C). The growth rate was calculated from the mean of 3 replicates using the equation y(t) = y0 * e (growth rate * t) (y = number of cells at final time point, y0 =

number of cells at initial time point, t = time). The mean cell doubling time from 3 replicates was calculated as doubling time = In(2)/growth rate.

2. Organoid passing and freezing

Gastric organoids were dissociated out of Matrigel gently using TrypLE Express incubation for 5 mins at 37°C. Following 5 ml of adDMEM/F12 +/+/+ containing 5% (vol/vol) FBS were added for inactivating the TrypLE. After centrifugation at 300g for 5 min, organoid fragments were resuspended in Matrigel and reseeded at ratios (1:3), allowing the formation of new ones. After pipetted out of Matrigel, organoids were stocked in 1.5ml cryotubes with recovery cell culture freezing medium containing 10% DMSO for organoids cryopreservation.

3. Genomic analysis

To evaluate the somatic mutations in the 9 paired tumor tissues and organoids lines, next-generation sequencing was performed with the Illumina HiSeq PE150 (Illumina, Inc., CA, USA) using a total of 0.6 μg genomic DNA per sample. Valid sequencing data were mapped to the reference human genome (UCSC hg19) by Burrows-Wheeler Aligner (BWA) software to get the initial mapping results stored in BAM format. Then, SAMtools and Picard (http://broadinstitute.github.io/picard/)were used to sort BAM files and do duplicate marking, local realignment, and base quality recalibration to generate the final BAM file for computation of the sequence coverage and depth.

4. H&E, Immunohistochemistry and Immunofluorescence

Gastric tissues and organoids were fixed in 4% paraformaldehyde (PFA) for 24h at room temperature (RT). Then the specimen was transferred to the Vacuum Tissue Processor Leica ASP200S (Leica Biosystems, Germany) for dehydration, and embedded into formalin-fixed paraffin-embedded (FFPE) blocks with tissue embedding center Leica EG1150 (Leica Biosystems, Germany). The FFPE blocks were then sectioned at 4-6 μm using a manual

rotary microtome Leica RM2235 (Leica Biosystems, Germany) and were stained with hematoxylin and eosin (H&E) solution. For immunostaining staining, the specimens were cut into 4-6μm thick sections, deparaffinized, and rehydrated and 3% hydrogen peroxide in methanol was used for the blockage of endogenous peroxidase at RT. Then, the sections were washed with phosphate-buffered saline (PBS; pH 7.2-7.6, three times), and the sections were washed with PBS again after heat mediated antigen retrieval was performed. Subsequently, the sections were incubated with primary antibodies overnight at 4˚C, including pan Cytokeratin (pan-CK,1:200 dilutions; #MA5-13203, Invitrogen, USA), carcinoembryonic antigen (CEA, 1:150 dilutions; #ZM-0062, Zhong Shan Golden Bridge Biological Technology Inc., Beijing, China) and caudal type homeobox 2 (CDX-2, 1:100 dilutions; #ZA-0520, Zhong Shan Golden Bridge Biological Technology Inc., Beijing, China). Then sections were washed with PBS and incubated with the respective secondary antibody (#kit-5020, Maixin, china). The slides were rinsed in PBS again, and treated with diaminobenzidine (DAB; 1:50) for 1-3 min, and finally counterstained with hematoxylin according to a standard protocol. Images were acquired on a Leica DM500 microscope (Leica Biosystems, Germany). For Immunofluorescence staining, organoids were harvested, fixed in eBioscience™ IC Fixation Buffer for 1h at 4 °C, and dehydrated with 30% sucrose overnight at 4 °C. After centrifugation and sucrose removal, organoids were mixed by optical coherence tomography (OCT), Then we placed the mixture into molds at -20°C for solidified. After samples were sectioned, we permeated them with eBioscience™ Permeabilization Buffer for 5 min and blocked them with 10% bovine serum albumin/PBS. They were incubated with primary antibodies, including Epithelial Cell Adhesion Molecule (EpCAM, 1:400 dilutions; #2929, Cell Signaling Technology, USA) and prominin-1 (CD133, 1:400 dilutions; #64326, Cell Signaling Technology, USA)overnight at 4 °C. Primary antibodies were detected by incubating with Alexa Fluor 488- (1:1000 dilutions; #ab150113, Abcam,

USA) and Alexa Fluor 568 (1:1000 dilutions; #175471, Abcam, USA) for 1h at RT. Nuclei were counterstained with DAPI (1ug/ml in Methanol, Sigma) for 5 min, and imaging was performed on a Confocal Laser Scanning Microscope Leica TCS SP8 (Leica, Germany).

5. Viability Assay

Gastric Organoids were harvested and dissociated following the passaging procedure described above. Cell pellets were resuspended in AdDMEM/F12+++ and were counted with the Cellometer ® Auto 2000. Then appropriate cells were diluted in Matrigel. 35 μl of medium/Matrigel containing 5000 cells were seeded onto pre-cooled 96-well plates (jingan# J09602). In addition, plates with medium/Matrigel (no cells) were served as the background control for the viability assay. And all plates were incubated for 15 min in a cell culture incubator. Gently add 100 μL of room temperature complete gastric organoid medium to each well. Three days post-seeding, media was removed and replaced by media containing six concentrations of 5-FU, oxaliplatin, irinotecan, and docetaxel. After three days, media were removed and replaced with 100 μl of organoid media containing 20% CellTiter-Blue ®Reagent (Promega#G8080, USA). The plates were agitated for 2 hours in a cell culture incubator prior to record fluorescence at 560/590 nm (Synergy H1MFD, Biotek, USA). The determination of IC50 values was conducted using Graph Pad Prism9.

6. Apoptosis assay

Organoids were treated with 1.5 μM docetaxel for 72 h. According to manufacturer instructions, cells were then isolated from Matrigel and stained with annexin V–FITC/PI (cat: KGA 105-DGA 108, KeyGEN BioTECH). After incubation for 30 min at 4°C, cells were analyzed using flow cytometry (FACS Canto; BD Biosciences).

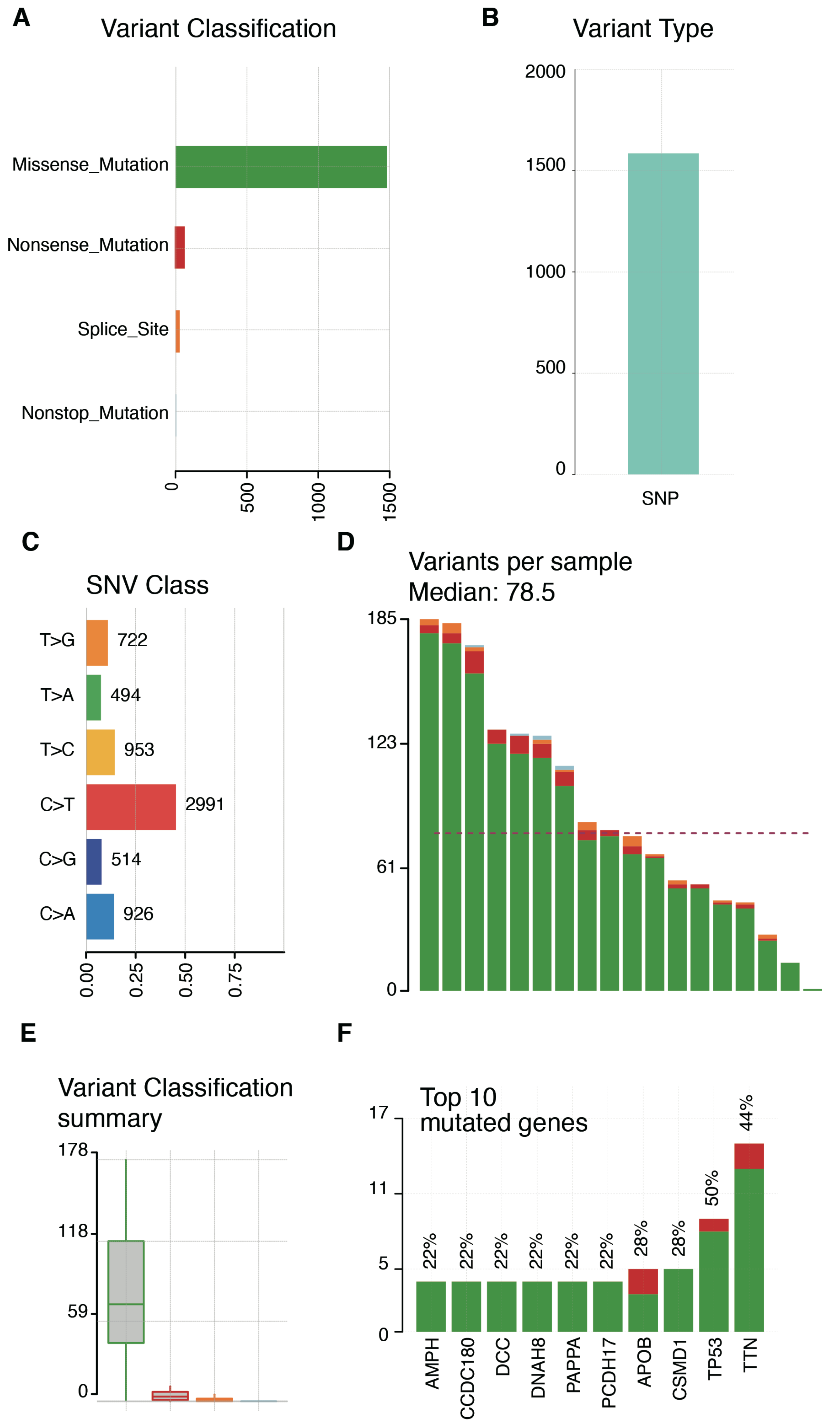
A
Variant Classification
Missense_Mutation
Nonsense_Mutation
Splice_Site
Nonstop_Mutation
0
500
1000
1500
B
Variant Type
2000
1500
1000
500
0
SNP
C
SNV Class
T>G 722
T>A 494
T>C 953
C>T 2991
C>G 514
C>A 926
0.00
0.25
0.50
0.75
D
Variants per sample
Median: 78.5
185
123
61
0
E
Variant Classification
summary
178
118
59
0
F
Top 10
mutated genes
17
11
5
0
22%
22%
22%
22%
22%
22%
28%
28%
50%
44%
AMPH
CCDC180
DCC
DNAH8
PAPPA
PCDH17
APOB
CSMD1
TP53
TTN

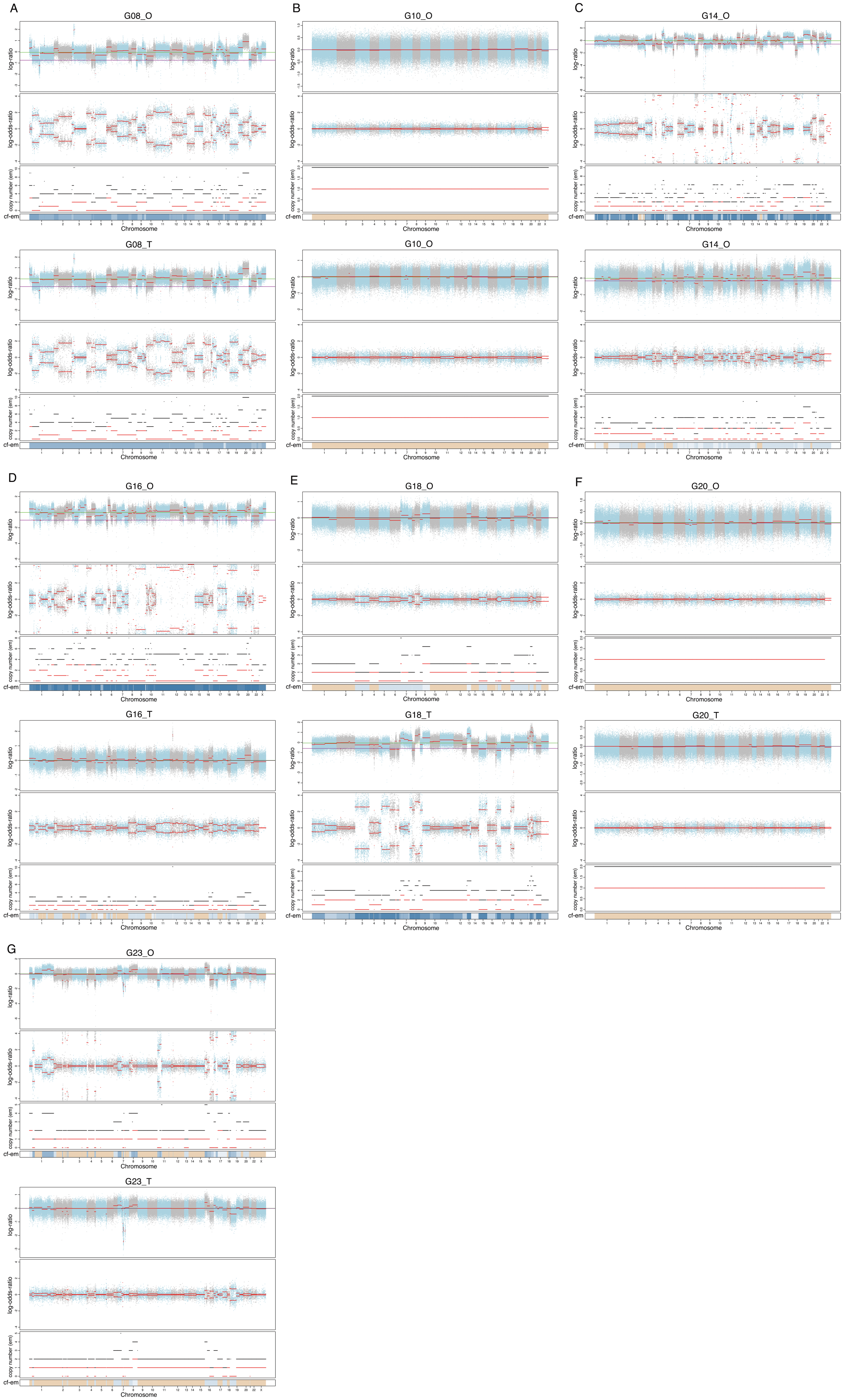
A
G08_O
G08_T
B
G10_O
G10_O
C
G14_O
G14_O
D
G16_O
G16_T
E
G18_O
G18_T
F
G20_O
G20_T
G
G23_O
G23_T
log-ratio
log-odds-ratio
copy number (em)
cf-em
Chromosome

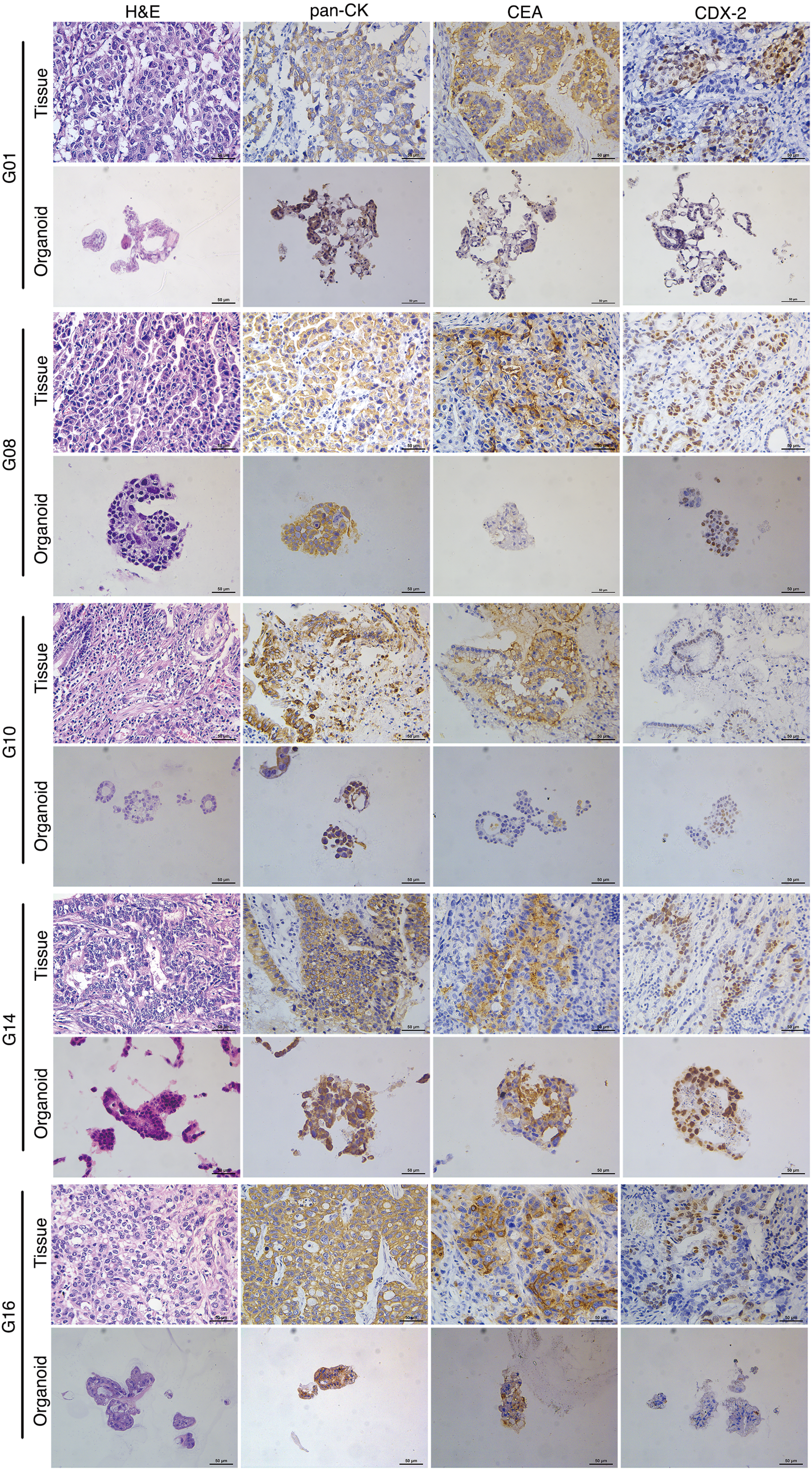

H&E
pan-CK
CEA
CDX-2
G01
Tissue
Organoid
G08
Tissue
Organoid
G10
Tissue
Organoid
G14
Tissue
Organoid
G16
Tissue
Organoid
50 μm

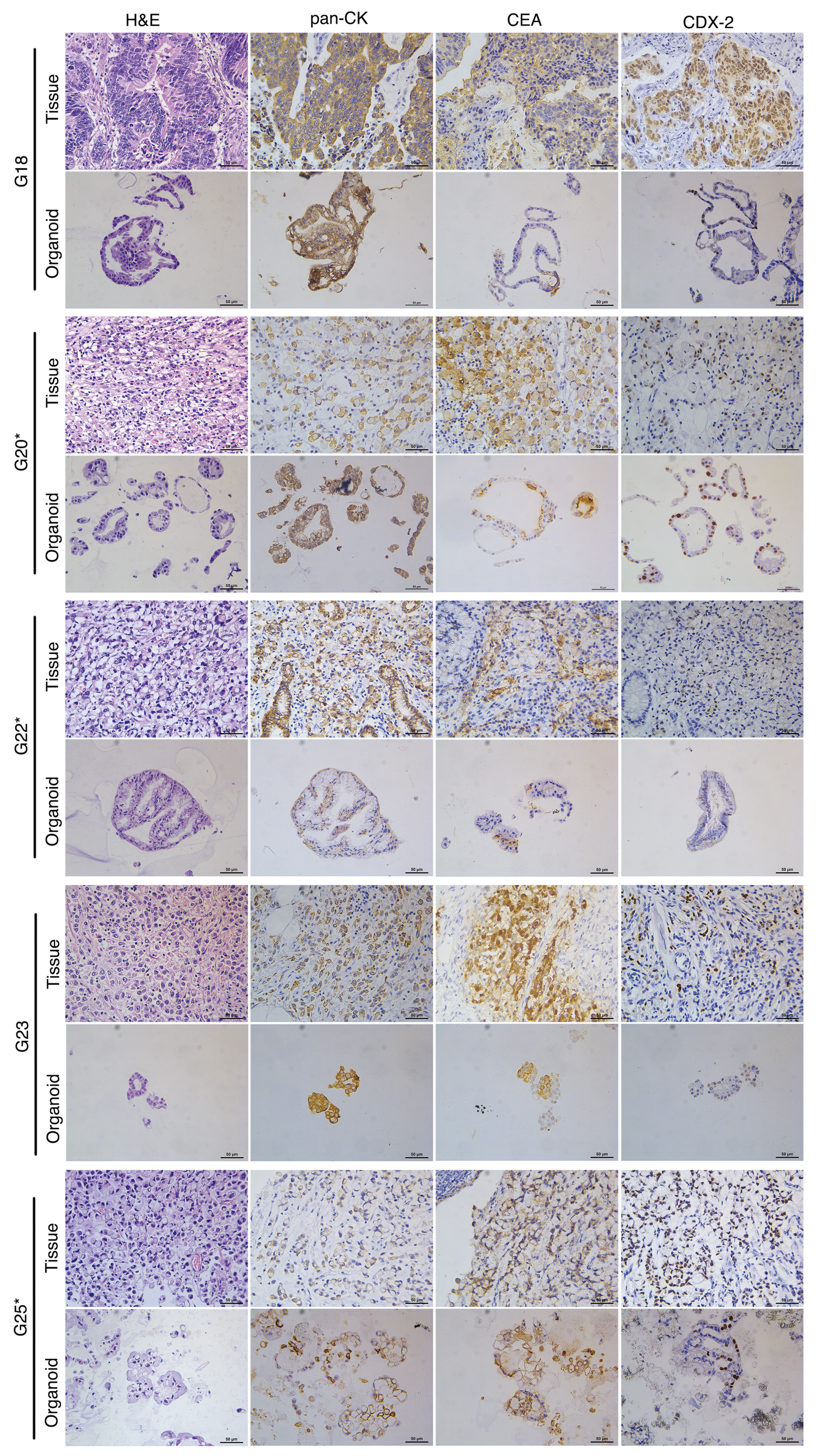

H&E
pan-CK
CEA
CDX-2
G18
G20*
G22*
G23
G25*
Tissue
Organoid
50 µm

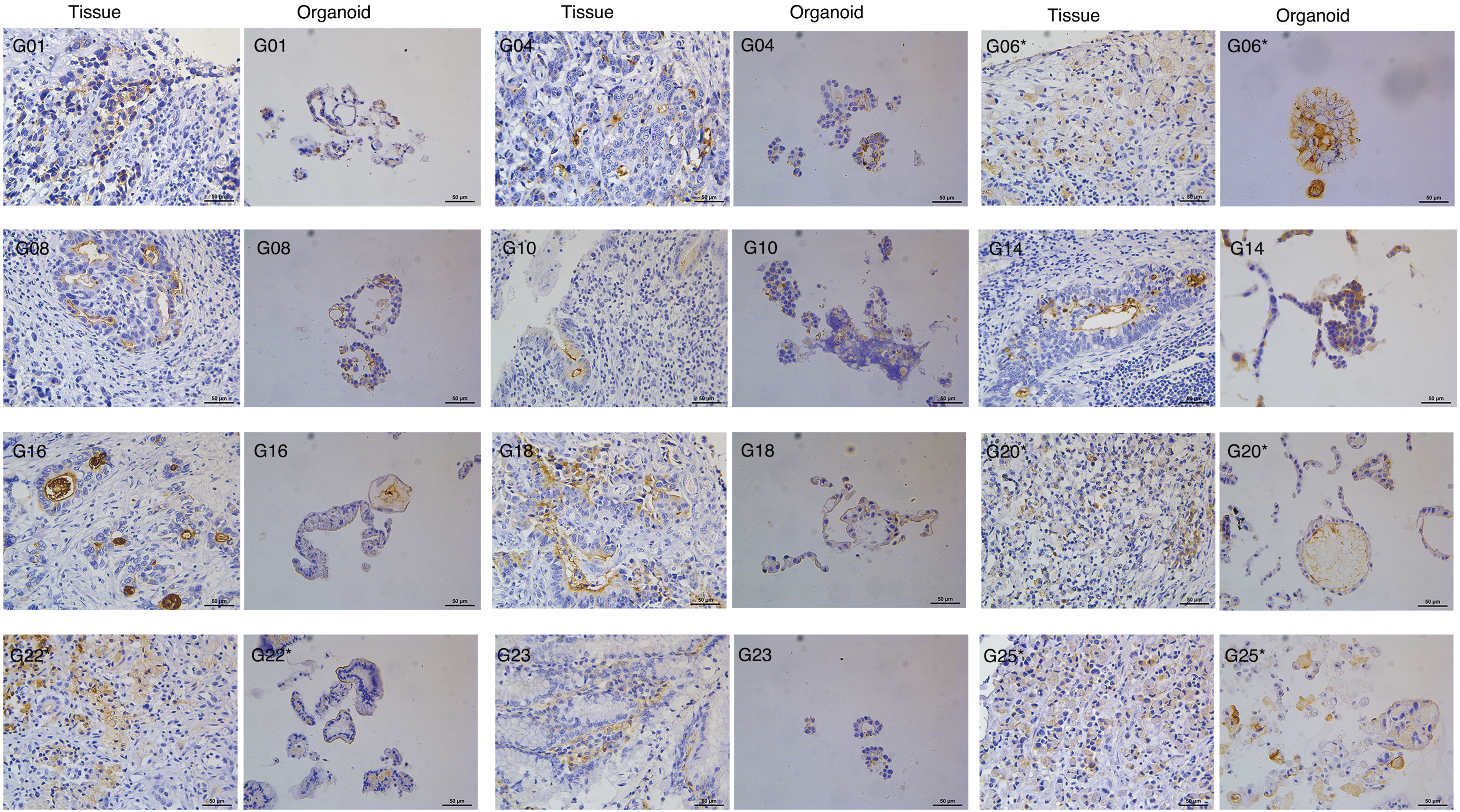
Tissue
Organoid
Tissue
Organoid
Tissue
Organoid
G01
G01
G04
G04
G06*
G06*
G08
G08
G10
G10
G14
G14
G16
G16
G18
G18
G20*
G20*
G22*
G22*
G23
G23
G25*
G25*

Supplementary Video 1 the growth status of G06(SRCC) every 3 hours for 72 hours

Supplementary Video 2 the growth status of G04(Non-SRCC) every 3 hours for 72 hours

Supplement Fig. 1 The basic information of SNV
A Summary of variant classification detected by deep targeted sequencing of all nine paired samples. **B** Variant type of all samples. **C** SNV class detected of all samples. **D** Variants per sample. **E** Variant classification summary of all samples. **F** Top 10 mutated genes of all samples.

Supplement Fig. 2 Genome-wide gene copy number variations (CNVs) of Organoids and paired primary tumors
A-G CNV analysis of G08_O, G08_T, G10_O, G10_T, G14_O, G14_T, G16_O, G16_T, G18_O, G18_T, G20_O, G20_T, G23_O, G23_T. O: organoids; T: original tissue. The top and middle diagrams show the distribution of logR and logOR values of all mutation sites in the chromosome. The bottom diagram is the copy number of the sample obtained by the CBS algorithm

Supplement Fig. 3 Histological characteristics of organoids and the primary tissue
H&E and IHC staining (pan-CK, CEA, and CDX-2) showed that the successfully cultured organoids (non-SRCC: G01, G08, G10, G14 and G16) and primary tumors are consistent in histological characteristics. Scale bars, 50 μm.

Supplement Fig. 4 Histological characteristics of organoids and the primary tissue
H&E and IHC staining (pan-CK, CEA, and CDX-2) showed that the successfully cultured organoids (SRCC: G20, G22 and G25, non-SRCC: G18 and G23) and primary tumors are consistent in histological characteristics. Scale bars, 50 μm. *, SRCC.

Supplement Fig. 5 The expression of CD133 in all organoids and the primary tissues
IHC staining (CD133) showed that the expression of CD133 of organoids and primary tumors were highly similar. Scale bars, 50 μm. *, SRCC.
