# Supplementary material for: Establishment of gastric signet ring cell carcinoma organoid for the therapeutic drug testing
Source: Cell Death Discov. 2022 Jan 10;8:6. doi: 10.1038/s41420-021-00803-7 (PMC8748936; doi:10.1038/s41420-021-00803-7)
Supplement: Supplementary file 5 — Supplementary Materials and Methods [file 41420_2021_803_MOESM5_ESM.docx]

**Supplementary Materials and Methods**

**1. Tumor cells isolation and establishment of human gastric organoid cultures**

Gastric cancer tissues were washed in the cold PBS with 1X penicillin/streptomycin (P/S) three times. Use scalpels and scissors to mince human gastric tissues into small pieces (1–5 mm^3^) in a 10-cm culture dish. Then, tissue was inserted into a gentleMACS C Tube with a volume of 5 ml digestion buffer (advanced DMEM/F12 containing 1X P/S, 2.5% FBS, 0.6mg/mL collagenases, 20mg/mL hyaluronidase and 10mM Y-27632). The C tube was then placed onto the gentleMACS Octo dissociator with heater. After tumor tissue dissociation, tissue debris were removed by passing the mixture through a 70uM cell strainer before centrifugation at 300g for 5 min at 4 °C. The pellets were resuspended in 10 ml adDMEM/F12 +/+/+ (adDMEM/F12 containing penicillin/streptomycin, 10 mM HEPES and 2 mM GlutaMAX) and centrifuged again at 300g for 5 min at 4 °C. If there were red blood cells precipitation, add 1-2 ml red blood cell lysis buffer to lyse at room temperature for 3 minutes, and add 10 ml AdDMEM/F12 +/+/+ before centrifugation at 300g for 5 min at 4 °C，then cells were counted with the Cellometer ® Auto 2000 and ViaStainTM AOPI Staining Solution (NexcelomBioscience, USA), and appropriate cell dilutions were done in cold Matrigel growth factor reduced basement membrane matrix(corning, 356321). 40ul drops (~20000 cells) were plated in the middle of one well of a pre-warmed 24-well plate at 37°C for 10-15 mins. After the Matrigel solidified, 400 ul of gastric organoid medium (advanced DMEM/ F12, 10 mM HEPES and 2 mM GlutaMAX, 100U/ml P/S, 1X B27 supplement, 1.25mM N-Acetylcysteine, 5mM Nicotinamide, 100ng/ml Noggin, 100ng/ml Wnt3a, 500ng/ml R-Spondin 1, 50ng/ml EGF, 25ng/ml EGF2, 200ng/ml FGF10, 2mM A83-01, 10mM Y-27632, 1nM Gastrin) was added to each well and plates transferred to the CO2 incubator (5% CO2, 37°C). The growth rate was calculated from the mean of 3 replicates using the equation y(t) = y0 * e (growth rate * t) (y = number of cells at ﬁnal time point, y0 = number of cells at initial time point, t = time). The mean cell doubling time from 3 replicates was calculated as doubling time = In(2)/growth rate.

**2. Organoid passing and freezing**

Gastric organoids were dissociated out of Matrigel gently using TrypLE Express incubation for 5 mins at 37°C. Following 5 ml of adDMEM/F12 +/+/+ containing 5% (vol/vol) FBS were added for inactivating the TrypLE. After centrifugation at 300g for 5 min, organoid fragments were resuspended in Matrigel and reseeded at ratios (1:3), allowing the formation of new ones. After pipetted out of Matrigel, organoids were stocked in 1.5ml cryotubes with recovery cell culture freezing medium containing 10% DMSO for organoids cryopreservation.

**3. Genomic analysis**

To evaluate the somatic mutations in the 9 paired tumor tissues and organoids lines, next-generation sequencing was performed with the Illumina HiSeq PE150 (Illumina, Inc., CA, USA) using a total of 0.6 μg genomic DNA per sample. Valid sequencing data were mapped to the reference human genome (UCSC hg19) by Burrows-Wheeler Aligner (BWA) software to get the initial mapping results stored in BAM format. Then, SAMtools and Picard (http://broadinstitute.github.io/picard/)were used to sort BAM files and do duplicate marking, local realignment, and base quality recalibration to generate the final BAM file for computation of the sequence coverage and depth.

**4. H&E, Immunohistochemistry and Immunofluorescence**

Gastric tissues and organoids were ﬁxed in 4% paraformaldehyde (PFA) for 24h at room temperature (RT). Then the specimen was transferred to the Vacuum Tissue Processor Leica ASP200S (Leica Biosystems, Germany) for dehydration, and embedded into formalin‑fixed paraffin‑embedded (FFPE) blocks with tissue embedding center Leica EG1150 (Leica Biosystems, Germany). The FFPE blocks were then sectioned at 4-6 μm using a manual rotary microtome Leica RM2235 (Leica Biosystems, Germany) and were stained with hematoxylin and eosin (H&E) solution. For immunostaining staining, the specimens were cut into 4‑6µm thick sections, deparaffinized, and rehydrated and 3% hydrogen peroxide in methanol was used for the blockage of endogenous peroxidase at RT. Then, the sections were washed with phosphate-buffered saline (PBS; pH 7.2-7.6, three times), and the sections were washed with PBS again after heat mediated antigen retrieval was performed. Subsequently, the sections were incubated with primary antibodies overnight at 4˚C, including pan Cytokeratin (pan-CK,1:200 dilutions; #MA5-13203, Invitrogen, USA), carcinoembryonic antigen (CEA, 1:150 dilutions; #ZM-0062, Zhong Shan Golden Bridge Biological Technology Inc., Beijing, China) and caudal type homeobox 2 (CDX-2, 1:100 dilutions; #ZA-0520, Zhong Shan Golden Bridge Biological Technology Inc., Beijing, China). Then sections were washed with PBS and incubated with the respective secondary antibody (#kit-5020, Maixin, china). The slides were rinsed in PBS again, and treated with diaminobenzidine (DAB; 1:50) for 1-3 min, and finally counterstained with hematoxylin according to a standard protocol. Images were acquired on a Leica DM500 microscope (Leica Biosystems, Germany). For Immunofluorescence staining, organoids were harvested, ﬁxed in eBioscience™ IC Fixation Buffer for 1h at 4 °C, and dehydrated with 30% sucrose overnight at 4 °C. After centrifugation and sucrose removal, organoids were mixed by optical coherence tomography (OCT), Then we placed the mixture into molds at -20°C for solidiﬁed. After samples were sectioned, we permeated them with eBioscience™ Permeabilization Buffer for 5 min and blocked them with 10% bovine serum albumin/PBS. They were incubated with primary antibodies, including Epithelial Cell Adhesion Molecule (EpCAM, 1:400 dilutions; #2929, Cell Signaling Technology, USA) and prominin-1 (CD133, 1:400 dilutions; #64326, Cell Signaling Technology, USA)overnight at 4 °C. Primary antibodies were detected by incubating with Alexa Fluor 488- (1:1000 dilutions; #ab150113, Abcam, USA) and Alexa Fluor 568 (1:1000 dilutions; #175471, Abcam, USA) for 1h at RT. Nuclei were counterstained with DAPI (1ug/ml in Methanol, Sigma) for 5 min, and imaging was performed on a Confocal Laser Scanning Microscope Leica TCS SP8 (Leica, Germany).

**5. Viability Assay**

Gastric Organoids were harvested and dissociated following the passaging procedure described above. Cell pellets were resuspended in AdDMEM/F12+++ and were counted with the Cellometer ® Auto 2000. Then appropriate cells were diluted in Matrigel. 35 µl of medium/Matrigel containing 5000 cells were seeded onto pre-cooled 96-well plates (jingan# J09602). In addition, plates with medium/Matrigel (no cells) were served as the background control for the viability assay. And all plates were incubated for 15 min in a cell culture incubator. Gently add 100 µL of room temperature complete gastric organoid medium to each well. Three days post-seeding, media was removed and replaced by media containing six concentrations of 5-FU, oxaliplatin, irinotecan, and docetaxel. After three days, media were removed and replaced with 100 µl of organoid media containing 20% CellTiter-Blue ®Reagent (Promega#G8080, USA). The plates were agitated for 2 hours in a cell culture incubator prior to record ﬂuorescence at 560/590 nm (Synergy H1MFD, Biotek, USA). The determination of IC50 values was conducted using Graph Pad Prism9.

**6.** **Apoptosis assay**

Organoids were treated with 1.5 µM docetaxel for 72 h. According to manufacturer instructions, cells were then isolated from Matrigel and stained with annexin V–FITC/PI (cat: KGA 105-DGA 108, KeyGEN BioTECH). After incubation for 30 min at 4°C, cells were analyzed using flow cytometry (FACS Canto; BD Biosciences).
