# Supplementary material for: Establishment of gastric signet ring cell carcinoma organoid for the therapeutic drug testing
Source: Cell Death Discov. 2022 Jan 10;8:6. doi: 10.1038/s41420-021-00803-7 (PMC8748936; doi:10.1038/s41420-021-00803-7)
Supplement: Supplementary file 11 — Supplement legends [file 41420_2021_803_MOESM11_ESM.docx]

Supplementary Video 1 the growth status of G06(SRCC) every 3 hours for 72 hours

Supplementary Video 2 the growth status of G04(Non-SRCC) every 3 hours for 72 hours

**Supplement Fig. 1** The basic information of SNV

**A** Summary of variant classification detected by deep targeted sequencing of all nine paired samples. **B** Variant type of all samples. **C** SNV class detected of all samples. **D** Variants per sample. **E** Variant classification summary of all samples. **F** Top 10 mutated genes of all samples.

**Supplement Fig. 2**  Genome-wide gene copy number variations (CNVs) of Organoids and paired primary tumors

**A-G** CNV analysis of G08_O, G08_T, G10_O, G10_T, G14_O, G14_T, G16_O, G16_T, G18_O, G18_T, G20_O, G20_T, G23_O, G23_T. O: organoids; T: original tissue. The top and middle diagrams show the distribution of logR and logOR values of all mutation sites in the chromosome. The bottom diagram is the copy number of the sample obtained by the CBS algorithm

**Supplement Fig. 3** Histological characteristics of organoids and the primary tissue

H&E and IHC staining (pan-CK, CEA, and CDX-2) showed that the successfully cultured organoids (non-SRCC: G01, G08, G10, G14 and G16) and primary tumors are consistent in histological characteristics. Scale bars, 50 μm.

**Supplement Fig. 4** Histological characteristics of organoids and the primary tissue

H&E and IHC staining (pan-CK, CEA, and CDX-2) showed that the successfully cultured organoids (SRCC: G20, G22 and G25, non-SRCC: G18 and G23) and primary tumors are consistent in histological characteristics. Scale bars, 50 μm. *, SRCC.

**Supplement Fig. 5** The expression of CD133 in all organoids and the primary tissues

IHC staining (CD133) showed that the expression of CD133 of organoids and primary tumors were highly similar. Scale bars, 50 μm. *, SRCC.
