# Supplementary material for: Establishment of gastric signet ring cell carcinoma organoid for the therapeutic drug testing
Source: Cell Death Discov. 2022 Jan 10;8:6. doi: 10.1038/s41420-021-00803-7 (PMC8748936; doi:10.1038/s41420-021-00803-7)
Supplement: Supplementary file 12 — Highlight [file 41420_2021_803_MOESM12_ESM.docx]

Highlight：

1. Gastric cancer organoid lines could be established by 3D culture.
2. Whether SRCC or Non-SRCC organoids maintain the primary tumorigenic character.
3. Gastric organoids can be used as a personalized drug screening model in vitro.
