# Supplementary material for: Establishment of gastric signet ring cell carcinoma organoid for the therapeutic drug testing
Source: Cell Death Discov. 2022 Jan 10;8:6. doi: 10.1038/s41420-021-00803-7 (PMC8748936; doi:10.1038/s41420-021-00803-7)
Supplement: Supplementary file 14 — Author Contributions Section [file 41420_2021_803_MOESM14_ESM.docx]

**Author Contributions Section**

WZ, SC and YT designed the overall study and revised the paper; GL and SM performed most of the experiments and the draft of the manuscript; QW performed data analysis; DK, ZY and ZG participated in data collection; LF and KZ provided technical and material support. All authors read and approved the final paper.
