# Supplementary material for: Establishment of gastric signet ring cell carcinoma organoid for the therapeutic drug testing
Source: Cell Death Discov. 2022 Jan 10;8:6. doi: 10.1038/s41420-021-00803-7 (PMC8748936; doi:10.1038/s41420-021-00803-7)
Supplement: Supplementary file 16 — Informed consent form [file 41420_2021_803_MOESM16_ESM.pdf]

进展期胃癌个体化治疗及预后评价可行性回顾性研究

知情同意书（INFORMED CONSENT FORM）

方案编号：**CH-GI-056**

申办单位：国家癌症中心中国医学科学院北京协和医学院肿瘤医院

主要研究者：田艳涛

尊敬的受试者：

我们邀请您参加一项临床研究：进展期胃癌个体化治疗及预后评价可行性回顾性研究(CH-GI-056)。在您决定是否参加这项研究之前，了解这项研究对您很重要。请仔细阅读以下信息，您可以与亲朋好友进行商量后做决定。如果您已经详细了解了这项研究，不再有疑问，并决定参加此项研究，您需要签署这份知情同意书。

一、研究背景

1、研究性质：本研究是由国家癌症中心/中国医学科学院北京协和医学院肿瘤医院（以下称：中国医学科学院肿瘤医院）发起的回顾性研究。

2、胃癌是全球第五大常见恶性肿瘤，也是导致全球癌症相关死亡的第二大原因。然而受制于研究模型的缺乏，胃癌发生机制、胃癌个性化治疗等研究进展缓慢。类器官技术是近十年来发展最为迅猛的一种体外培养技术，曾被誉为2013年度最为重要的科学进展之一。类器官是干细胞在体外三维环境中培养所形成的细胞结构，通过体外模拟细胞在体内的生存环境，从而能够高效培养原代细胞，包括正常细胞、肿瘤细胞。因此，胃癌组织来源的类器官作为新一代临床前肿瘤模型，在模拟胃癌的发生与发展、抗肿瘤药物研发与筛选、肿瘤靶向治疗与个体化医疗等方面都有良好的应用前景。

此项研究已经得到中国医学科学院肿瘤医院伦理委员会的审批，伦理委员会是一个保护受试者权益的组织。在您决定参加本临床研究之前，建议您参考本《知情同意书》，结合主管医生的说明，以便帮助您更好的理解。

二、研究目的

以局部进展期胃腺癌患者为研究对象，探索进展期胃癌个体化治疗方案及预后评价体系。

三、研究对象

1、本项研究拟纳入来自于中国医学科学院肿瘤医院的100名受试者；

2、入组标准：（1）18 岁＜年龄＜75 岁；（2）胃原发病灶经内镜活检组织病理学诊断为胃腺癌（乳头状腺癌 pap、管状腺癌 tub、黏液腺癌 muc、印戒细胞癌 sig、低分化腺癌 por)；（3）术前临

床分期为 cT2-4a，N-/+，根据 AJCC-8th TNM 肿瘤分期；（4）术前检查肿瘤无直接侵犯胰腺、脾脏及其他临近器官；（5）术前 ECOG 体力状态评分 0/1；（6）术前 ASA 评分 I-III；（7）患者知情同意。

3、排除标准：（1）妊娠或哺乳期妇女；（2）患有严重精神疾病；（3）胃手术史（包括针对胃癌的 ESD/EMR）；（4）5 年内有其他恶性疾病史；（5）6 个月内有不稳定性心绞痛或心肌梗塞史；（6）6 个月内有脑梗塞或脑出血病史；（7）需要进行其他肿瘤的同期手术治疗患者；（8）继发性胃癌肿瘤患者；（9）合并严重基础疾病不能耐受手术者。

四、研究步骤

1、如果您自愿参加此研究，由护士采集您在术后的离体的癌组织及癌旁组织，这些标本的使用不会影响您正常的诊断和治疗。同时您将授权中国医学科学院肿瘤医院基于科学研究目的查阅您在本次研究中的医疗资料和检测结果。

2、当我们收集好这些样本后，将建立专门的样本资料库，数据库内容包括“样本编号” 、“参与者病案号”，两者会同时被标记在样本管上，以便实现参加者信息、样本号的“双重保险”，确保在数据分析时的信息准确性。这些样本将送到本院病因及癌变研究室进行胃癌类器官培养，剩余生物样本将返还中国医学科学院肿瘤医院保存。

五、可能的风险和受益

1、可能的风险：本项研究为非干预性研究，不会影响和干扰您的常规医疗诊断和治疗，因此不会额外增加您的风险。整个研究过程接受中国医学科学院肿瘤医院伦理委员会的监督，研究过程中如果您遇到任何疑问可以向研究医生或伦理委员会咨询。

2、可能的受益：由于此项研究为非干预性研究，其结果不一定能够直接用于您的诊断和治疗，但是通过对您的样本进行类器官培养或医疗数据进行分析将有助于未来对肿瘤疾病做出明确的诊断或进行有效的治疗，并提高肿瘤的治愈率。在此我们为您能够参与科学研究，并为医学的发展做出贡献表示感谢！

六、自愿原则

参与本项研究是完全自愿的，您可以随时退出而不需要理由，您不参加研究或中途退出研究，的都不会影响您和医务人员的关系及对您疾病的诊治。如果您决定参加本项研究，在研究过程中您的医生将会把可能影响您身体状况，或影响您决定是否继续参加研究的有关信息告知您。

七、研究费用

标本检测费用由研究者/申办方承担，参加此项研究不会额外增加您的费用。

八、联系人及联系方式

如果您本人对此项研究有任何疑问，可直接与中国医学科学院肿瘤医院联系，联系人：田艳涛、解亦斌、马帅医生，联系电话：010-87787120。

如果您有与受试者/参与者权利相关的任何问题，或者您想反映参与本研究过程中遭遇的困难、不满和忧虑，或者想提供与本研究有关的意见和建议，请联系中国医学科学院肿瘤医院伦理委员会，联系电话：010-87788495，电子邮件：cancergcp@163.com。

九、隐私保密原则

本项研究中您的信息和医学资料将在法律要求的范围内保密。我们将采用个人身份资料去识别处理来保护您的隐私：您在入组后使用项目统一编号，您的个人信息和医疗资料由您的医生或其研究团队收集，数据将会被编码、储存并受保护，使用者看到的仅为个体编号，不再接触您的姓名等资料。您有权随时询问记录的信息内容并要求更正错误。

您的研究医生将对您的数据进行分析，并可能从您的医学记录中进一步收集本项研究需要的其他数据。研究期间或之后的任何时间，在法律允许的范围内，伦理委员会或政府管理部门的成员按相应权限可以查阅您的个人资料，以便对研究数据的真实性、准确性和可靠性进行核实。研究结果以统计分析后的数据形式发表，不包含任何可识别的患者信息。

十、研究终止

参加研究期间，您可以随时退出研究而不需要理由，您的决定不会对您继续接受医学治疗产生任何影响。当您退出研究或研究终止时，研究医生将与您讨论后续的诊疗措施。

进展期胃癌个体化治疗及预后评价可行性回顾性研究

知情同意书签字页

受试者声明

研究医生已经向我详细解释了这项研究的目的、过程，以及参加研究可能存在的风险和益处；我已经仔细阅读了知情同意书，所有问题得到了满意的解答，并且我已经全部理解。

我同意我的研究医生收集和使用我的医学资料。我同意中国医学科学院肿瘤医院基于科学研究目的查阅本项研究中我的医学资料和研究结果。我同意伦理委员会的成员以及政府管理部门的代表，在遵循保密原则的前提下，按相应权限查阅我的医学资料，我了解查看这些记录是为了确保从这项研究中收集的资料是真实、完整和可靠的。

我明确：我参加本项研究是自愿的，我可以在任何时候退出研究并且不会对我之后的医学治疗或合法权益产生影响。

我已经得到一份已签署的知情同意书的副本。签署这份同意书的时候，我没有放弃任何我的合法权益。

我同意参加本项研究中肿瘤组织/血液等生物标记物检查：

是（请在方格内打勾）☑

否（请在方格内打勾）☐

患者签名 [illegible] 日期 [illegible]

法定代理人签名*________ 日期________ 与患者的关系________

***除非受试者不能阅读（如文盲或盲人）或因其他原因不能自行签名，否则不需要法定代理人的签名**

研究者声明

我承诺在临床研究中严格遵守 GCP 原则、遵守国家和中国医学科学院肿瘤医院的相关规定，保护受试者权益，保证研究工作时间和研究数据的真实、完整、可靠。我同意中国医学科学院肿瘤医院基于科学研究目的查阅本项研究的医学资料和研究结果。我同意将研究剩余的生物标本返还中国医学科学院肿瘤医院保存。

我已经向患者充分解释了本项研究的目的、过程，以及参加研究可能存在的风险和益处，患者已经获得了足够的信息来做出参加本项研究的决定。我将为患者或其法定代理人提供一个署名并注明日期的知情同意书副本。

研究者签名[illegible] 日期 2020.8.21

研究者联系方式 010-87787120

进展期胃癌个体化治疗及预后评价可行性回顾性研究

知情同意书签字页

受试者声明

研究医生已经向我详细解释了这项研究的目的、过程，以及参加研究可能存在的风险和益处；我已经仔细阅读了知情同意书，所有问题得到了满意的解答，并且我已经全部理解。

我同意我的研究医生收集和使用我的医学资料。我同意中国医学科学院肿瘤医院基于科学研究目的查阅本项研究中我的医学资料和研究结果。我同意伦理委员会的成员以及政府管理部门的代表，在遵循保密原则的前提下，按相应权限查阅我的医学资料，我了解查看这些记录是为了确保从这项研究中收集的资料是真实、完整和可靠的。

我明确：我参加本项研究是自愿的，我可以在任何时候退出研究并且不会对我之后的医学治疗或合法权益产生影响。

我已经得到一份已签署的知情同意书的副本。签署这份同意书的时候，我没有放弃任何我的合法权益。

我同意参加本项研究中肿瘤组织/血液等生物标记物检查：

是（请在方格内打勾）☑

否（请在方格内打勾）☐

患者签名 刘福义 日期 2020 8.15

法定代理人签名*________ 日期________ 与患者的关系________

*除非受试者不能阅读（如文盲或盲人）或因其他原因不能自行签名，否则不需要法定代理人的签名

研究者声明

我承诺在临床研究中严格遵守 GCP 原则、遵守国家和中国医学科学院肿瘤医院的相关规定，保护受试者权益，保证研究工作时间和研究数据的真实、完整、可靠。我同意中国医学科学院肿瘤医院基于科学研究目的查阅本项研究的医学资料和研究结果。我同意将研究剩余的生物标本返还中国医学科学院肿瘤医院保存。

我已经向患者充分解释了本项研究的目的、过程，以及参加研究可能存在的风险和益处，患者已经获得了足够的信息来做出参加本项研究的决定。我将为患者或其法定代理人提供一个署名并注明日期的知情同意书副本。

研究者签名 [illegible] 日期 2020.08.15

研究者联系方式 010-87787120

进展期胃癌个体化治疗及预后评价可行性回顾性研究

知情同意书签字页

受试者声明

研究医生已经向我详细解释了这项研究的目的、过程，以及参加研究可能存在的风险和益处；我已经仔细阅读了知情同意书，所有问题得到了满意的解答，并且我已经全部理解。

我同意我的研究医生收集和使用我的医学资料。我同意中国医学科学院肿瘤医院基于科学研究目的查阅本项研究中我的医学资料和研究结果。我同意伦理委员会的成员以及政府管理部门的代表，在遵循保密原则的前提下，按相应权限查阅我的医学资料，我了解查看这些记录是为了确保从这项研究中收集的资料是真实、完整和可靠的。

我明确：我参加本项研究是自愿的，我可以在任何时候退出研究并且不会对我之后的医学治疗或合法权益产生影响。

我已经得到一份已签署的知情同意书的副本。签署这份同意书的时候，我没有放弃任何我的合法权益。

我同意参加本项研究中肿瘤组织/血液等生物标记物检查：

是（请在方格内打勾）☑

否（请在方格内打勾）□

患者签名 王[illegible]之 日期 2020.8.8

法定代理人签名*__________ 日期__________ 与患者的关系__________

*除非受试者不能阅读（如文盲或盲人）或因其他原因不能自行签名，否则不需要法定代理人的签名

研究者声明

我承诺在临床研究中严格遵守 GCP 原则、遵守国家和中国医学科学院肿瘤医院的相关规定，保护受试者权益，保证研究工作时间和研究数据的真实、完整、可靠。我同意中国医学科学院肿瘤医院基于科学研究目的查阅本项研究的医学资料和研究结果。我同意将研究剩余的生物标本返还中国医学科学院肿瘤医院保存。

我已经向患者充分解释了本项研究的目的、过程，以及参加研究可能存在的风险和益处，患者已经获得了足够的信息来做出参加本项研究的决定。我将为患者或其法定代理人提供一个署名并注明日期的知情同意书副本。

研究者签名 [illegible] 日期 2020.8.8

研究者联系方式 010-87787120

进展期胃癌个体化治疗及预后评价可行性回顾性研究

知情同意书签字页

受试者声明

研究医生已经向我详细解释了这项研究的目的、过程，以及参加研究可能存在的风险和益处；我已经仔细阅读了知情同意书，所有问题得到了满意的解答，并且我已经全部理解。

我同意我的研究医生收集和使用我的医学资料。我同意中国医学科学院肿瘤医院基于科学研究目的查阅本项研究中我的医学资料和研究结果。我同意伦理委员会的成员以及政府管理部门的代表，在遵循保密原则的前提下，按相应权限查阅我的医学资料，我了解查看这些记录是为了确保从这项研究中收集的资料是真实、完整和可靠的。

我明确：我参加本项研究是自愿的，我可以在任何时候退出研究并且不会对我之后的医学治疗或合法权益产生影响。

我已经得到一份已签署的知情同意书的副本。签署这份同意书的时候，我没有放弃任何我的合法权益。

我同意参加本项研究中肿瘤组织/血液等生物标记物检查：

是（请在方格内打勾）☑

否（请在方格内打勾）☐

患者签名 姬新月 日期 2020.8.22

法定代理人签名*________ 日期________ 与患者的关系________

*除非受试者不能阅读（如文盲或盲人）或因其他原因不能自行签名，否则不需要法定代理人的签名

研究者声明

我承诺在临床研究中严格遵守 GCP 原则、遵守国家和中国医学科学院肿瘤医院的相关规定，保护受试者权益，保证研究工作时间和研究数据的真实、完整、可靠。我同意中国医学科学院肿瘤医院基于科学研究目的查阅本项研究的医学资料和研究结果。我同意将研究剩余的生物标本返还中国医学科学院肿瘤医院保存。

我已经向患者充分解释了本项研究的目的、过程，以及参加研究可能存在的风险和益处，患者已经获得了足够的信息来做出参加本项研究的决定。我将为患者或其法定代理人提供一个署名并注明日期的知情同意书副本。

研究者签名[illegible]日期2020.8.22

研究者联系方式010-87787120

进展期胃癌个体化治疗及预后评价可行性回顾性研究

知情同意书签字页

受试者声明

研究医生已经向我详细解释了这项研究的目的、过程，以及参加研究可能存在的风险和益处；我已经仔细阅读了知情同意书，所有问题得到了满意的解答，并且我已经全部理解。

我同意我的研究医生收集和使用我的医学资料。我同意中国医学科学院肿瘤医院基于科学研究目的查阅本项研究中我的医学资料和研究结果。我同意伦理委员会的成员以及政府管理部门的代表，在遵循保密原则的前提下，按相应权限查阅我的医学资料，我了解查看这些记录是为了确保从这项研究中收集的资料是真实、完整和可靠的。

我明确：我参加本项研究是自愿的，我可以在任何时候退出研究并且不会对我之后的医学治疗或合法权益产生影响。

我已经得到一份已签署的知情同意书的副本。签署这份同意书的时候，我没有放弃任何我的合法权益。

我同意参加本项研究中肿瘤组织/血液等生物标记物检查：

是（请在方格内打勾）☑

否（请在方格内打勾）□

患者签名 胡[illegible] 日期 2020.8.29

法定代理人签名*________ 日期________ 与患者的关系________

*除非受试者不能阅读（如文盲或盲人）或因其他原因不能自行签名，否则不需要法定代理人的签名

研究者声明

我承诺在临床研究中严格遵守 GCP 原则、遵守国家和中国医学科学院肿瘤医院的相关规定，保护受试者权益，保证研究工作时间和研究数据的真实、完整、可靠。我同意中国医学科学院肿瘤医院基于科学研究目的查阅本项研究的医学资料和研究结果。我同意将研究剩余的生物标本返还中国医学科学院肿瘤医院保存。

我已经向患者充分解释了本项研究的目的、过程，以及参加研究可能存在的风险和益处，患者已经获得了足够的信息来做出参加本项研究的决定。我将为患者或其法定代理人提供一个署名并注明日期的知情同意书副本。

研究者签名[illegible] 日期 2020.8.29

研究者联系方式 010-87787120

进展期胃癌个体化治疗及预后评价可行性回顾性研究

知情同意书签字页

受试者声明

研究医生已经向我详细解释了这项研究的目的、过程，以及参加研究可能存在的风险和益处；我已经仔细阅读了知情同意书，所有问题得到了满意的解答，并且我已经全部理解。

我同意我的研究医生收集和使用我的医学资料。我同意中国医学科学院肿瘤医院基于科学研究目的查阅本项研究中我的医学资料和研究结果。我同意伦理委员会的成员以及政府管理部门的代表，在遵循保密原则的前提下，按相应权限查阅我的医学资料，我了解查看这些记录是为了确保从这项研究中收集的资料是真实、完整和可靠的。

我明确：我参加本项研究是自愿的，我可以在任何时候退出研究并且不会对我之后的医学治疗或合法权益产生影响。

我已经得到一份已签署的知情同意书的副本。签署这份同意书的时候，我没有放弃任何我的合法权益。

我同意参加本项研究中肿瘤组织/血液等生物标记物检查：

是（请在方格内打勾）☑

否（请在方格内打勾）□

患者签名 李兴好 日期 2020.8.30

法定代理人签名*______________ 日期______________ 与患者的关系______________

*除非受试者不能阅读（如文盲或盲人）或因其他原因不能自行签名，否则不需要法定代理人的签名

研究者声明

我承诺在临床研究中严格遵守 GCP 原则、遵守国家和中国医学科学院肿瘤医院的相关规定，保护受试者权益，保证研究工作时间和研究数据的真实、完整、可靠。我同意中国医学科学院肿瘤医院基于科学研究目的查阅本项研究的医学资料和研究结果。我同意将研究剩余的生物标本返还中国医学科学院肿瘤医院保存。

我已经向患者充分解释了本项研究的目的、过程，以及参加研究可能存在的风险和益处，患者已经获得了足够的信息来做出参加本项研究的决定。我将为患者或其法定代理人提供一个署名并注明日期的知情同意书副本。

研究者签名__[illegible]__日期__2020.8.30__

研究者联系方式__010-87787120__

进展期胃癌个体化治疗及预后评价可行性回顾性研究

知情同意书签字页

受试者声明

研究医生已经向我详细解释了这项研究的目的、过程，以及参加研究可能存在的风险和益处；我已经仔细阅读了知情同意书，所有问题得到了满意的解答，并且我已经全部理解。

我同意我的研究医生收集和使用我的医学资料。我同意中国医学科学院肿瘤医院基于科学研究目的查阅本项研究中我的医学资料和研究结果。我同意伦理委员会的成员以及政府管理部门的代表，在遵循保密原则的前提下，按相应权限查阅我的医学资料，我了解查看这些记录是为了确保从这项研究中收集的资料是真实、完整和可靠的。

我明确：我参加本项研究是自愿的，我可以在任何时候退出研究并且不会对我之后的医学治疗或合法权益产生影响。

我已经得到一份已签署的知情同意书的副本。签署这份同意书的时候，我没有放弃任何我的合法权益。

我同意参加本项研究中肿瘤组织/血液等生物标记物检查：

是（请在方格内打勾）☑

否（请在方格内打勾）□

患者签名 薛利云 日期 2020.8.31

法定代理人签名*__________ 日期__________ 与患者的关系__________

*除非受试者不能阅读（如文盲或盲人）或因其他原因不能自行签名，否则不需要法定代理人的签名

研究者声明

我承诺在临床研究中严格遵守 GCP 原则、遵守国家和中国医学科学院肿瘤医院的相关规定，保护受试者权益，保证研究工作时间和研究数据的真实、完整、可靠。我同意中国医学科学院肿瘤医院基于科学研究目的查阅本项研究的医学资料和研究结果。我同意将研究剩余的生物标本返还中国医学科学院肿瘤医院保存。

我已经向患者充分解释了本项研究的目的、过程，以及参加研究可能存在的风险和益处，患者已经获得了足够的信息来做出参加本项研究的决定。我将为患者或其法定代理人提供一个署名并注明日期的知情同意书副本。

研究者签名[illegible] 日期 2020.8.31

研究者联系方式 01087787120

进展期胃癌个体化治疗及预后评价可行性回顾性研究

知情同意书签字页

受试者声明

研究医生已经向我详细解释了这项研究的目的、过程，以及参加研究可能存在的风险和益处；我已经仔细阅读了知情同意书，所有问题得到了满意的解答，并且我已经全部理解。

我同意我的研究医生收集和使用我的医学资料。我同意中国医学科学院肿瘤医院基于科学研究目的查阅本项研究中我的医学资料和研究结果。我同意伦理委员会的成员以及政府管理部门的代表，在遵循保密原则的前提下，按相应权限查阅我的医学资料，我了解查看这些记录是为了确保从这项研究中收集的资料是真实、完整和可靠的。

我明确：我参加本项研究是自愿的，我可以在任何时候退出研究并且不会对我之后的医学治疗或合法权益产生影响。

我已经得到一份已签署的知情同意书的副本。签署这份同意书的时候，我没有放弃任何我的合法权益。

我同意参加本项研究中肿瘤组织/血液等生物标记物检查：

是（请在方格内打勾）☑

否（请在方格内打勾）□

患者签名 张建民 日期 2020.8.31

法定代理人签名*________ 日期________ 与患者的关系________

*除非受试者不能阅读（如文盲或盲人）或因其他原因不能自行签名，否则不需要法定代理人的签名

研究者声明

我承诺在临床研究中严格遵守 GCP 原则、遵守国家和中国医学科学院肿瘤医院的相关规定，保护受试者权益，保证研究工作时间和研究数据的真实、完整、可靠。我同意中国医学科学院肿瘤医院基于科学研究目的查阅本项研究的医学资料和研究结果。我同意将研究剩余的生物标本返还中国医学科学院肿瘤医院保存。

我已经向患者充分解释了本项研究的目的、过程，以及参加研究可能存在的风险和益处，患者已经获得了足够的信息来做出参加本项研究的决定。我将为患者或其法定代理人提供一个署名并注明日期的知情同意书副本。

研究者签名 [illegible] 日期 2020.8.31

研究者联系方式 010-87787120.

进展期胃癌个体化治疗及预后评价可行性回顾性研究

知情同意书签字页

受试者声明

研究医生已经向我详细解释了这项研究的目的、过程，以及参加研究可能存在的风险和益处；我已经仔细阅读了知情同意书，所有问题得到了满意的解答，并且我已经全部理解。

我同意我的研究医生收集和使用我的医学资料。我同意中国医学科学院肿瘤医院基于科学研究目的查阅本项研究中我的医学资料和研究结果。我同意伦理委员会的成员以及政府管理部门的代表，在遵循保密原则的前提下，按相应权限查阅我的医学资料，我了解查看这些记录是为了确保从这项研究中收集的资料是真实、完整和可靠的。

我明确：我参加本项研究是自愿的，我可以在任何时候退出研究并且不会对我之后的医学治疗或合法权益产生影响。

我已经得到一份已签署的知情同意书的副本。签署这份同意书的时候，我没有放弃任何我的合法权益。

我同意参加本项研究中肿瘤组织/血液等生物标记物检查：

是（请在方格内打勾）☑

否（请在方格内打勾）□

患者签名 刘玉玲 日期 2020.9.2

法定代理人签名*__________ 日期__________ 与患者的关系__________

*除非受试者不能阅读（如文盲或盲人）或因其他原因不能自行签名，否则不需要法定代理人的签名

研究者声明

我承诺在临床研究中严格遵守 GCP 原则、遵守国家和中国医学科学院肿瘤医院的相关规定，保护受试者权益，保证研究工作时间和研究数据的真实、完整、可靠。我同意中国医学科学院肿瘤医院基于科学研究目的查阅本项研究的医学资料和研究结果。我同意将研究剩余的生物标本返还中国医学科学院肿瘤医院保存。

我已经向患者充分解释了本项研究的目的、过程，以及参加研究可能存在的风险和益处，患者已经获得了足够的信息来做出参加本项研究的决定。我将为患者或其法定代理人提供一个署名并注明日期的知情同意书副本。

研究者签名[illegible] 日期 2020.9.2

研究者联系方式 010-87787120

进展期胃癌个体化治疗及预后评价可行性回顾性研究
知情同意书签字页

受试者声明

研究医生已经向我详细解释了这项研究的目的、过程，以及参加研究可能存在的风险和益处；我已经仔细阅读了知情同意书，所有问题得到了满意的解答，并且我已经全部理解。

我同意我的研究医生收集和使用我的医学资料。我同意中国医学科学院肿瘤医院基于科学研究目的查阅本项研究中我的医学资料和研究结果。我同意伦理委员会的成员以及政府管理部门的代表，在遵循保密原则的前提下，按相应权限查阅我的医学资料，我了解查看这些记录是为了确保从这项研究中收集的资料是真实、完整和可靠的。

我明确：我参加本项研究是自愿的，我可以在任何时候退出研究并且不会对我之后的医学治疗或合法权益产生影响。

我已经得到一份已签署的知情同意书的副本。签署这份同意书的时候，我没有放弃任何我的合法权益。

我同意参加本项研究中肿瘤组织/血液等生物标记物检查：

是（请在方格内打勾）☑

否（请在方格内打勾）□

患者签名 [illegible] 日期 2020.9.7

法定代理人签名*__________ 日期__________ 与患者的关系__________

*除非受试者不能阅读（如文盲或盲人）或因其他原因不能自行签名，否则不需要法定代理人的签名

研究者声明

我承诺在临床研究中严格遵守 GCP 原则、遵守国家和中国医学科学院肿瘤医院的相关规定，保护受试者权益，保证研究工作时间和研究数据的真实、完整、可靠。我同意中国医学科学院肿瘤医院基于科学研究目的查阅本项研究的医学资料和研究结果。我同意将研究剩余的生物标本返还中国医学科学院肿瘤医院保存。

我已经向患者充分解释了本项研究的目的、过程，以及参加研究可能存在的风险和益处，患者已经获得了足够的信息来做出参加本项研究的决定。我将为患者或其法定代理人提供一个署名并注明日期的知情同意书副本。

研究者签名[illegible] 日期 2020.9.7

研究者联系方式 01087787120

进展期胃癌个体化治疗及预后评价可行性回顾性研究

知情同意书签字页

受试者声明

研究医生已经向我详细解释了这项研究的目的、过程，以及参加研究可能存在的风险和益处；我已经仔细阅读了知情同意书，所有问题得到了满意的解答，并且我已经全部理解。

我同意我的研究医生收集和使用我的医学资料。我同意中国医学科学院肿瘤医院基于科学研究目的查阅本项研究中我的医学资料和研究结果。我同意伦理委员会的成员以及政府管理部门的代表，在遵循保密原则的前提下，按相应权限查阅我的医学资料，我了解查看这些记录是为了确保从这项研究中收集的资料是真实、完整和可靠的。

我明确：我参加本项研究是自愿的，我可以在任何时候退出研究并且不会对我之后的医学治疗或合法权益产生影响。

我已经得到一份已签署的知情同意书的副本。签署这份同意书的时候，我没有放弃任何我的合法权益。

我同意参加本项研究中肿瘤组织/血液等生物标记物检查：

是（请在方格内打勾）☑

否（请在方格内打勾）□

患者签名何青春 日期2020.9.5

法定代理人签名*____________ 日期____________ 与患者的关系____________

*除非受试者不能阅读（如文盲或盲人）或因其他原因不能自行签名，否则不需要法定代理人的签名

研究者声明

我承诺在临床研究中严格遵守 GCP 原则、遵守国家和中国医学科学院肿瘤医院的相关规定，保护受试者权益，保证研究工作时间和研究数据的真实、完整、可靠。我同意中国医学科学院肿瘤医院基于科学研究目的查阅本项研究的医学资料和研究结果。我同意将研究剩余的生物标本返还中国医学科学院肿瘤医院保存。

我已经向患者充分解释了本项研究的目的、过程，以及参加研究可能存在的风险和益处，患者已经获得了足够的信息来做出参加本项研究的决定。我将为患者或其法定代理人提供一个署名并注明日期的知情同意书副本。

研究者签名[illegible]日期2020.9.5

研究者联系方式010-87787120

进展期胃癌个体化治疗及预后评价可行性回顾性研究

知情同意书签字页

受试者声明

研究医生已经向我详细解释了这项研究的目的、过程，以及参加研究可能存在的风险和益处；我已经仔细阅读了知情同意书，所有问题得到了满意的解答，并且我已经全部理解。

我同意我的研究医生收集和使用我的医学资料。我同意中国医学科学院肿瘤医院基于科学研究目的查阅本项研究中我的医学资料和研究结果。我同意伦理委员会的成员以及政府管理部门的代表，在遵循保密原则的前提下，按相应权限查阅我的医学资料，我了解查看这些记录是为了确保从这项研究中收集的资料是真实、完整和可靠的。

我明确：我参加本项研究是自愿的，我可以在任何时候退出研究并且不会对我之后的医学治疗或合法权益产生影响。

我已经得到一份已签署的知情同意书的副本。签署这份同意书的时候，我没有放弃任何我的合法权益。

我同意参加本项研究中肿瘤组织/血液等生物标记物检查：

是（请在方格内打勾） ☑

否（请在方格内打勾） □

患者签名 [illegible] 日期 2020.9.9

法定代理人签名*________ 日期________ 与患者的关系________

*除非受试者不能阅读（如文盲或盲人）或因其他原因不能自行签名，否则不需要法定代理人的签名

研究者声明

我承诺在临床研究中严格遵守 GCP 原则、遵守国家和中国医学科学院肿瘤医院的相关规定，保护受试者权益，保证研究工作时间和研究数据的真实、完整、可靠。我同意中国医学科学院肿瘤医院基于科学研究目的查阅本项研究的医学资料和研究结果。我同意将研究剩余的生物标本返还中国医学科学院肿瘤医院保存。

我已经向患者充分解释了本项研究的目的、过程，以及参加研究可能存在的风险和益处，患者已经获得了足够的信息来做出参加本项研究的决定。我将为患者或其法定代理人提供一个署名并注明日期的知情同意书副本。

研究者签名 [illegible] 日期 2020.9.9

研究者联系方式 010-87787120

进展期胃癌个体化治疗及预后评价可行性回顾性研究

知情同意书签字页

受试者声明

研究医生已经向我详细解释了这项研究的目的、过程，以及参加研究可能存在的风险和益处；我已经仔细阅读了知情同意书，所有问题得到了满意的解答，并且我已经全部理解。

我同意我的研究医生收集和使用我的医学资料。我同意中国医学科学院肿瘤医院基于科学研究目的查阅本项研究中我的医学资料和研究结果。我同意伦理委员会的成员以及政府管理部门的代表，在遵循保密原则的前提下，按相应权限查阅我的医学资料，我了解查看这些记录是为了确保从这项研究中收集的资料是真实、完整和可靠的。

我明确：我参加本项研究是自愿的，我可以在任何时候退出研究并且不会对我之后的医学治疗或合法权益产生影响。

我已经得到一份已签署的知情同意书的副本。签署这份同意书的时候，我没有放弃任何我的合法权益。

我同意参加本项研究中肿瘤组织/血液等生物标记物检查：

是（请在方格内打勾）☑

否（请在方格内打勾）□

患者签名 李廷军 日期 2020.9.13

法定代理人签名*________ 日期________ 与患者的关系________

*除非受试者不能阅读（如文盲或盲人）或因其他原因不能自行签名，否则不需要法定代理人的签名

研究者声明

我承诺在临床研究中严格遵守 GCP 原则、遵守国家和中国医学科学院肿瘤医院的相关规定，保护受试者权益，保证研究工作时间和研究数据的真实、完整、可靠。我同意中国医学科学院肿瘤医院基于科学研究目的查阅本项研究的医学资料和研究结果。我同意将研究剩余的生物标本返还中国医学科学院肿瘤医院保存。

我已经向患者充分解释了本项研究的目的、过程，以及参加研究可能存在的风险和益处，患者已经获得了足够的信息来做出参加本项研究的决定。我将为患者或其法定代理人提供一个署名并注明日期的知情同意书副本。

研究者签名 [illegible] 日期 2020.9.13

研究者联系方式 010.87787120

进展期胃癌个体化治疗及预后评价可行性回顾性研究

知情同意书签字页

受试者声明

研究医生已经向我详细解释了这项研究的目的、过程，以及参加研究可能存在的风险和益处；我已经仔细阅读了知情同意书，所有问题得到了满意的解答，并且我已经全部理解。

我同意我的研究医生收集和使用我的医学资料。我同意中国医学科学院肿瘤医院基于科学研究目的查阅本项研究中我的医学资料和研究结果。我同意伦理委员会的成员以及政府管理部门的代表，在遵循保密原则的前提下，按相应权限查阅我的医学资料，我了解查看这些记录是为了确保从这项研究中收集的资料是真实、完整和可靠的。

我明确：我参加本项研究是自愿的，我可以在任何时候退出研究并且不会对我之后的医学治疗或合法权益产生影响。

我已经得到一份已签署的知情同意书的副本。签署这份同意书的时候，我没有放弃任何我的合法权益。

我同意参加本项研究中肿瘤组织/血液等生物标记物检查：

是（请在方格内打勾）☑

否（请在方格内打勾）☐

患者签名 徐水悦 日期 2020.9.13

法定代理人签名*__________ 日期__________ 与患者的关系__________

*除非受试者不能阅读（如文盲或盲人）或因其他原因不能自行签名，否则不需要法定代理人的签名

研究者声明

我承诺在临床研究中严格遵守 GCP 原则、遵守国家和中国医学科学院肿瘤医院的相关规定，保护受试者权益，保证研究工作时间和研究数据的真实、完整、可靠。我同意中国医学科学院肿瘤医院基于科学研究目的查阅本项研究的医学资料和研究结果。我同意将研究剩余的生物标本返还中国医学科学院肿瘤医院保存。

我已经向患者充分解释了本项研究的目的、过程，以及参加研究可能存在的风险和益处，患者已经获得了足够的信息来做出参加本项研究的决定。我将为患者或其法定代理人提供一个署名并注明日期的知情同意书副本。

研究者签名[illegible] 日期 2020.9.13

研究者联系方式 010-87787120

进展期胃癌个体化治疗及预后评价可行性回顾性研究

知情同意书签字页

受试者声明

研究医生已经向我详细解释了这项研究的目的、过程，以及参加研究可能存在的风险和益处；我已经仔细阅读了知情同意书，所有问题得到了满意的解答，并且我已经全部理解。

我同意我的研究医生收集和使用我的医学资料。我同意中国医学科学院肿瘤医院基于科学研究目的查阅本项研究中我的医学资料和研究结果。我同意伦理委员会的成员以及政府管理部门的代表，在遵循保密原则的前提下，按相应权限查阅我的医学资料，我了解查看这些记录是为了确保从这项研究中收集的资料是真实、完整和可靠的。

我明确：我参加本项研究是自愿的，我可以在任何时候退出研究并且不会对我之后的医学治疗或合法权益产生影响。

我已经得到一份已签署的知情同意书的副本。签署这份同意书的时候，我没有放弃任何我的合法权益。

我同意参加本项研究中肿瘤组织/血液等生物标记物检查：

是（请在方格内打勾）☑

否（请在方格内打勾）□

患者签名 王一珩 日期 2020.9.13

法定代理人签名*________ 日期________ 与患者的关系________

*除非受试者不能阅读（如文盲或盲人）或因其他原因不能自行签名，否则不需要法定代理人的签名

研究者声明

我承诺在临床研究中严格遵守 GCP 原则、遵守国家和中国医学科学院肿瘤医院的相关规定，保护受试者权益，保证研究工作时间和研究数据的真实、完整、可靠。我同意中国医学科学院肿瘤医院基于科学研究目的查阅本项研究的医学资料和研究结果。我同意将研究剩余的生物标本返还中国医学科学院肿瘤医院保存。

我已经向患者充分解释了本项研究的目的、过程，以及参加研究可能存在的风险和益处，患者已经获得了足够的信息来做出参加本项研究的决定。我将为患者或其法定代理人提供一个署名并注明日期的知情同意书副本。

研究者签名 [illegible] 日期 2020.9.13

研究者联系方式 010-87787120

进展期胃癌个体化治疗及预后评价可行性回顾性研究

知情同意书签字页

受试者声明

研究医生已经向我详细解释了这项研究的目的、过程，以及参加研究可能存在的风险和益处；我已经仔细阅读了知情同意书，所有问题得到了满意的解答，并且我已经全部理解。

我同意我的研究医生收集和使用我的医学资料。我同意中国医学科学院肿瘤医院基于科学研究目的查阅本项研究中我的医学资料和研究结果。我同意伦理委员会的成员以及政府管理部门的代表，在遵循保密原则的前提下，按相应权限查阅我的医学资料，我了解查看这些记录是为了确保从这项研究中收集的资料是真实、完整和可靠的。

我明确：我参加本项研究是自愿的，我可以在任何时候退出研究并且不会对我之后的医学治疗或合法权益产生影响。

我已经得到一份已签署的知情同意书的副本。签署这份同意书的时候，我没有放弃任何我的合法权益。

我同意参加本项研究中肿瘤组织/血液等生物标记物检查：

是（请在方格内打勾）☑

否（请在方格内打勾）☐

患者签名[illegible] 日期 2020.9.14

法定代理人签名*________ 日期________ 与患者的关系________

*除非受试者不能阅读（如文盲或盲人）或因其他原因不能自行签名，否则不需要法定代理人的签名

研究者声明

我承诺在临床研究中严格遵守 GCP 原则、遵守国家和中国医学科学院肿瘤医院的相关规定，保护受试者权益，保证研究工作时间和研究数据的真实、完整、可靠。我同意中国医学科学院肿瘤医院基于科学研究目的查阅本项研究的医学资料和研究结果。我同意将研究剩余的生物标本返还中国医学科学院肿瘤医院保存。

我已经向患者充分解释了本项研究的目的、过程，以及参加研究可能存在的风险和益处，患者已经获得了足够的信息来做出参加本项研究的决定。我将为患者或其法定代理人提供一个署名并注明日期的知情同意书副本。

研究者签名 [illegible] 日期 2020.9.14

研究者联系方式 010-87787120

进展期胃癌个体化治疗及预后评价可行性回顾性研究

知情同意书签字页

受试者声明

研究医生已经向我详细解释了这项研究的目的、过程，以及参加研究可能存在的风险和益处；我已经仔细阅读了知情同意书，所有问题得到了满意的解答，并且我已经全部理解。

我同意我的研究医生收集和使用我的医学资料。我同意中国医学科学院肿瘤医院基于科学研究目的查阅本项研究中我的医学资料和研究结果。我同意伦理委员会的成员以及政府管理部门的代表，在遵循保密原则的前提下，按相应权限查阅我的医学资料，我了解查看这些记录是为了确保从这项研究中收集的资料是真实、完整和可靠的。

我明确：我参加本项研究是自愿的，我可以在任何时候退出研究并且不会对我之后的医学治疗或合法权益产生影响。

我已经得到一份已签署的知情同意书的副本。签署这份同意书的时候，我没有放弃任何我的合法权益。

我同意参加本项研究中肿瘤组织/血液等生物标记物检查：

是（请在方格内打勾）☑

否（请在方格内打勾）☐

患者签名[illegible]　日期2020.9.15

法定代理人签名*____________日期____________与患者的关系____________

*除非受试者不能阅读（如文盲或盲人）或因其他原因不能自行签名，否则不需要法定代理人的签名

研究者声明

我承诺在临床研究中严格遵守 GCP 原则、遵守国家和中国医学科学院肿瘤医院的相关规定，保护受试者权益，保证研究工作时间和研究数据的真实、完整、可靠。我同意中国医学科学院肿瘤医院基于科学研究目的查阅本项研究的医学资料和研究结果。我同意将研究剩余的生物标本返还中国医学科学院肿瘤医院保存。

我已经向患者充分解释了本项研究的目的、过程，以及参加研究可能存在的风险和益处，患者已经获得了足够的信息来做出参加本项研究的决定。我将为患者或其法定代理人提供一个署名并注明日期的知情同意书副本。

研究者签名 [illegible] 日期 2020.9.15

研究者联系方式 010-87787120

进展期胃癌个体化治疗及预后评价可行性回顾性研究

知情同意书签字页

受试者声明

研究医生已经向我详细解释了这项研究的目的、过程，以及参加研究可能存在的风险和益处；我已经仔细阅读了知情同意书，所有问题得到了满意的解答，并且我已经全部理解。

我同意我的研究医生收集和使用我的医学资料。我同意中国医学科学院肿瘤医院基于科学研究目的查阅本项研究中我的医学资料和研究结果。我同意伦理委员会的成员以及政府管理部门的代表，在遵循保密原则的前提下，按相应权限查阅我的医学资料，我了解查看这些记录是为了确保从这项研究中收集的资料是真实、完整和可靠的。

我明确：我参加本项研究是自愿的，我可以在任何时候退出研究并且不会对我之后的医学治疗或合法权益产生影响。

我已经得到一份已签署的知情同意书的副本。签署这份同意书的时候，我没有放弃任何我的合法权益。

我同意参加本项研究中肿瘤组织/血液等生物标记物检查：

是（请在方格内打勾）☑

否（请在方格内打勾）□

患者签名 王俊林 日期 2020.9.16

法定代理人签名*__________ 日期__________ 与患者的关系__________

*除非受试者不能阅读（如文盲或盲人）或因其他原因不能自行签名，否则不需要法定代理人的签名

研究者声明

我承诺在临床研究中严格遵守 GCP 原则、遵守国家和中国医学科学院肿瘤医院的相关规定，保护受试者权益，保证研究工作时间和研究数据的真实、完整、可靠。我同意中国医学科学院肿瘤医院基于科学研究目的查阅本项研究的医学资料和研究结果。我同意将研究剩余的生物标本返还中国医学科学院肿瘤医院保存。

我已经向患者充分解释了本项研究的目的、过程，以及参加研究可能存在的风险和益处，患者已经获得了足够的信息来做出参加本项研究的决定。我将为患者或其法定代理人提供一个署名并注明日期的知情同意书副本。

研究者签名 [illegible] 日期 2020.9.16

研究者联系方式 010-87787120

进展期胃癌个体化治疗及预后评价可行性回顾性研究

知情同意书签字页

受试者声明

研究医生已经向我详细解释了这项研究的目的、过程，以及参加研究可能存在的风险和益处；我已经仔细阅读了知情同意书，所有问题得到了满意的解答，并且我已经全部理解。

我同意我的研究医生收集和使用我的医学资料。我同意中国医学科学院肿瘤医院基于科学研究目的查阅本项研究中我的医学资料和研究结果。我同意伦理委员会的成员以及政府管理部门的代表，在遵循保密原则的前提下，按相应权限查阅我的医学资料，我了解查看这些记录是为了确保从这项研究中收集的资料是真实、完整和可靠的。

我明确：我参加本项研究是自愿的，我可以在任何时候退出研究并且不会对我之后的医学治疗或合法权益产生影响。

我已经得到一份已签署的知情同意书的副本。签署这份同意书的时候，我没有放弃任何我的合法权益。

我同意参加本项研究中肿瘤组织/血液等生物标记物检查：

是（请在方格内打勾）☑

否（请在方格内打勾）☐

患者签名 [illegible] 日期 2020.9.15

法定代理人签名*________ 日期________ 与患者的关系________

*除非受试者不能阅读（如文盲或盲人）或因其他原因不能自行签名，否则不需要法定代理人的签名

研究者声明

我承诺在临床研究中严格遵守 GCP 原则、遵守国家和中国医学科学院肿瘤医院的相关规定，保护受试者权益，保证研究工作时间和研究数据的真实、完整、可靠。我同意中国医学科学院肿瘤医院基于科学研究目的查阅本项研究的医学资料和研究结果。我同意将研究剩余的生物标本返还中国医学科学院肿瘤医院保存。

我已经向患者充分解释了本项研究的目的、过程，以及参加研究可能存在的风险和益处，患者已经获得了足够的信息来做出参加本项研究的决定。我将为患者或其法定代理人提供一个署名并注明日期的知情同意书副本。

研究者签名 [illegible] 日期 2020.9.15

研究者联系方式 010-87787120

进展期胃癌个体化治疗及预后评价可行性回顾性研究

知情同意书签字页

受试者声明

研究医生已经向我详细解释了这项研究的目的、过程，以及参加研究可能存在的风险和益处；我已经仔细阅读了知情同意书，所有问题得到了满意的解答，并且我已经全部理解。

我同意我的研究医生收集和使用我的医学资料。我同意中国医学科学院肿瘤医院基于科学研究目的查阅本项研究中我的医学资料和研究结果。我同意伦理委员会的成员以及政府管理部门的代表，在遵循保密原则的前提下，按相应权限查阅我的医学资料，我了解查看这些记录是为了确保从这项研究中收集的资料是真实、完整和可靠的。

我明确：我参加本项研究是自愿的，我可以在任何时候退出研究并且不会对我之后的医学治疗或合法权益产生影响。

我已经得到一份已签署的知情同意书的副本。签署这份同意书的时候，我没有放弃任何我的合法权益。

我同意参加本项研究中肿瘤组织/血液等生物标记物检查：

是（请在方格内打勾）☑

否（请在方格内打勾）□

患者签名[illegible] 日期 2020.9.20

法定代理人签名*__________ 日期__________ 与患者的关系__________

*除非受试者不能阅读（如文盲或盲人）或因其他原因不能自行签名，否则不需要法定代理人的签名

研究者声明

我承诺在临床研究中严格遵守 GCP 原则、遵守国家和中国医学科学院肿瘤医院的相关规定，保护受试者权益，保证研究工作时间和研究数据的真实、完整、可靠。我同意中国医学科学院肿瘤医院基于科学研究目的查阅本项研究的医学资料和研究结果。我同意将研究剩余的生物标本返还中国医学科学院肿瘤医院保存。

我已经向患者充分解释了本项研究的目的、过程，以及参加研究可能存在的风险和益处，患者已经获得了足够的信息来做出参加本项研究的决定。我将为患者或其法定代理人提供一个署名并注明日期的知情同意书副本。

研究者签名[illegible]日期2020.9.20

研究者联系方式010-87787120

进展期胃癌个体化治疗及预后评价可行性回顾性研究

知情同意书签字页

受试者声明

研究医生已经向我详细解释了这项研究的目的、过程，以及参加研究可能存在的风险和益处；我已经仔细阅读了知情同意书，所有问题得到了满意的解答，并且我已经全部理解。

我同意我的研究医生收集和使用我的医学资料。我同意中国医学科学院肿瘤医院基于科学研究目的查阅本项研究中我的医学资料和研究结果。我同意伦理委员会的成员以及政府管理部门的代表，在遵循保密原则的前提下，按相应权限查阅我的医学资料，我了解查看这些记录是为了确保从这项研究中收集的资料是真实、完整和可靠的。

我明确：我参加本项研究是自愿的，我可以在任何时候退出研究并且不会对我之后的医学治疗或合法权益产生影响。

我已经得到一份已签署的知情同意书的副本。签署这份同意书的时候，我没有放弃任何我的合法权益。

我同意参加本项研究中肿瘤组织/血液等生物标记物检查：

是（请在方格内打勾）☑

否（请在方格内打勾）☐

患者签名 张霞 日期 2020.9.23

法定代理人签名*________ 日期________ 与患者的关系________

***除非受试者不能阅读（如文盲或盲人）或因其他原因不能自行签名，否则不需要法定代理人的签名**

研究者声明

我承诺在临床研究中严格遵守 GCP 原则、遵守国家和中国医学科学院肿瘤医院的相关规定，保护受试者权益，保证研究工作时间和研究数据的真实、完整、可靠。我同意中国医学科学院肿瘤医院基于科学研究目的查阅本项研究的医学资料和研究结果。我同意将研究剩余的生物标本返还中国医学科学院肿瘤医院保存。

我已经向患者充分解释了本项研究的目的、过程，以及参加研究可能存在的风险和益处，患者已经获得了足够的信息来做出参加本项研究的决定。我将为患者或其法定代理人提供一个署名并注明日期的知情同意书副本。

研究者签名 [illegible] 日期 2020.9.23

研究者联系方式 010-87787120

进展期胃癌个体化治疗及预后评价可行性回顾性研究

知情同意书签字页

受试者声明

研究医生已经向我详细解释了这项研究的目的、过程，以及参加研究可能存在的风险和益处；我已经仔细阅读了知情同意书，所有问题得到了满意的解答，并且我已经全部理解。

我同意我的研究医生收集和使用我的医学资料。我同意中国医学科学院肿瘤医院基于科学研究目的查阅本项研究中我的医学资料和研究结果。我同意伦理委员会的成员以及政府管理部门的代表，在遵循保密原则的前提下，按相应权限查阅我的医学资料，我了解查看这些记录是为了确保从这项研究中收集的资料是真实、完整和可靠的。

我明确：我参加本项研究是自愿的，我可以在任何时候退出研究并且不会对我之后的医学治疗或合法权益产生影响。

我已经得到一份已签署的知情同意书的副本。签署这份同意书的时候，我没有放弃任何我的合法权益。

我同意参加本项研究中肿瘤组织/血液等生物标记物检查：

是（请在方格内打勾）☑

否（请在方格内打勾）☐

患者签名 [illegible] 日期 2020.9.25

法定代理人签名*________ 日期________ 与患者的关系________

*除非受试者不能阅读（如文盲或盲人）或因其他原因不能自行签名，否则不需要法定代理人的签名

研究者声明

我承诺在临床研究中严格遵守 GCP 原则、遵守国家和中国医学科学院肿瘤医院的相关规定，保护受试者权益，保证研究工作时间和研究数据的真实、完整、可靠。我同意中国医学科学院肿瘤医院基于科学研究目的查阅本项研究的医学资料和研究结果。我同意将研究剩余的生物标本返还中国医学科学院肿瘤医院保存。

我已经向患者充分解释了本项研究的目的、过程，以及参加研究可能存在的风险和益处，患者已经获得了足够的信息来做出参加本项研究的决定。我将为患者或其法定代理人提供一个署名并注明日期的知情同意书副本。

研究者签名[illegible]日期 2020.9.25

研究者联系方式 010-87787120

进展期胃癌个体化治疗及预后评价可行性回顾性研究

知情同意书签字页

受试者声明

研究医生已经向我详细解释了这项研究的目的、过程，以及参加研究可能存在的风险和益处；我已经仔细阅读了知情同意书，所有问题得到了满意的解答，并且我已经全部理解。

我同意我的研究医生收集和使用我的医学资料。我同意中国医学科学院肿瘤医院基于科学研究目的查阅本项研究中我的医学资料和研究结果。我同意伦理委员会的成员以及政府管理部门的代表，在遵循保密原则的前提下，按相应权限查阅我的医学资料，我了解查看这些记录是为了确保从这项研究中收集的资料是真实、完整和可靠的。

我明确：我参加本项研究是自愿的，我可以在任何时候退出研究并且不会对我之后的医学治疗或合法权益产生影响。

我已经得到一份已签署的知情同意书的副本。签署这份同意书的时候，我没有放弃任何我的合法权益。

我同意参加本项研究中肿瘤组织/血液等生物标记物检查：

是（请在方格内打勾）☑

否（请在方格内打勾）□

患者签名 刘宏杰 日期 2020.9.27

法定代理人签名*________ 日期________ 与患者的关系________

*除非受试者不能阅读（如文盲或盲人）或因其他原因不能自行签名，否则不需要法定代理人的签名

研究者声明

我承诺在临床研究中严格遵守 GCP 原则、遵守国家和中国医学科学院肿瘤医院的相关规定，保护受试者权益，保证研究工作时间和研究数据的真实、完整、可靠。我同意中国医学科学院肿瘤医院基于科学研究目的查阅本项研究的医学资料和研究结果。我同意将研究剩余的生物标本返还中国医学科学院肿瘤医院保存。

我已经向患者充分解释了本项研究的目的、过程，以及参加研究可能存在的风险和益处，患者已经获得了足够的信息来做出参加本项研究的决定。我将为患者或其法定代理人提供一个署名并注明日期的知情同意书副本。

研究者签名[illegible]日期2020.9.27

研究者联系方式010-8778[illegible]

进展期胃癌个体化治疗及预后评价可行性回顾性研究

知情同意书签字页

受试者声明

研究医生已经向我详细解释了这项研究的目的、过程，以及参加研究可能存在的风险和益处；我已经仔细阅读了知情同意书，所有问题得到了满意的解答，并且我已经全部理解。

我同意我的研究医生收集和使用我的医学资料。我同意中国医学科学院肿瘤医院基于科学研究目的查阅本项研究中我的医学资料和研究结果。我同意伦理委员会的成员以及政府管理部门的代表，在遵循保密原则的前提下，按相应权限查阅我的医学资料，我了解查看这些记录是为了确保从这项研究中收集的资料是真实、完整和可靠的。

我明确：我参加本项研究是自愿的，我可以在任何时候退出研究并且不会对我之后的医学治疗或合法权益产生影响。

我已经得到一份已签署的知情同意书的副本。签署这份同意书的时候，我没有放弃任何我的合法权益。

我同意参加本项研究中肿瘤组织/血液等生物标记物检查：

是（请在方格内打勾）☑

否（请在方格内打勾）□

患者签名 赵兰春 日期 2020.10.11

法定代理人签名*__________ 日期__________ 与患者的关系__________

*除非受试者不能阅读（如文盲或盲人）或因其他原因不能自行签名，否则不需要法定代理人的签名

研究者声明

我承诺在临床研究中严格遵守 GCP 原则、遵守国家和中国医学科学院肿瘤医院的相关规定，保护受试者权益，保证研究工作时间和研究数据的真实、完整、可靠。我同意中国医学科学院肿瘤医院基于科学研究目的查阅本项研究的医学资料和研究结果。我同意将研究剩余的生物标本返还中国医学科学院肿瘤医院保存。

我已经向患者充分解释了本项研究的目的、过程，以及参加研究可能存在的风险和益处，患者已经获得了足够的信息来做出参加本项研究的决定。我将为患者或其法定代理人提供一个署名并注明日期的知情同意书副本。

研究者签名[illegible]日期2020.6.11

研究者联系方式010-87787120

进展期胃癌个体化治疗及预后评价可行性回顾性研究

知情同意书签字页

受试者声明

研究医生已经向我详细解释了这项研究的目的、过程，以及参加研究可能存在的风险和益处；我已经仔细阅读了知情同意书，所有问题得到了满意的解答，并且我已经全部理解。

我同意我的研究医生收集和使用我的医学资料。我同意中国医学科学院肿瘤医院基于科学研究目的查阅本项研究中我的医学资料和研究结果。我同意伦理委员会的成员以及政府管理部门的代表，在遵循保密原则的前提下，按相应权限查阅我的医学资料，我了解查看这些记录是为了确保从这项研究中收集的资料是真实、完整和可靠的。

我明确：我参加本项研究是自愿的，我可以在任何时候退出研究并且不会对我之后的医学治疗或合法权益产生影响。

我已经得到一份已签署的知情同意书的副本。签署这份同意书的时候，我没有放弃任何我的合法权益。

我同意参加本项研究中肿瘤组织/血液等生物标记物检查：

是（请在方格内打勾）☑

否（请在方格内打勾）□

患者签名 陈永利 日期 2021.01.09

法定代理人签名*______ 日期______ 与患者的关系______

*除非受试者不能阅读（如文盲或盲人）或因其他原因不能自行签名，否则不需要法定代理人的签名

研究者声明

我承诺在临床研究中严格遵守 GCP 原则、遵守国家和中国医学科学院肿瘤医院的相关规定，保护受试者权益，保证研究工作时间和研究数据的真实、完整、可靠。我同意中国医学科学院肿瘤医院基于科学研究目的查阅本项研究的医学资料和研究结果。我同意将研究剩余的生物标本返还中国医学科学院肿瘤医院保存。

我已经向患者充分解释了本项研究的目的、过程，以及参加研究可能存在的风险和益处，患者已经获得了足够的信息来做出参加本项研究的决定。我将为患者或其法定代理人提供一个署名并注明日期的知情同意书副本。

研究者签名[illegible]日期 2021.1.9

研究者联系方式 010-87787120

进展期胃癌个体化治疗及预后评价可行性回顾性研究

知情同意书签字页

受试者声明

研究医生已经向我详细解释了这项研究的目的、过程，以及参加研究可能存在的风险和益处；我已经仔细阅读了知情同意书，所有问题得到了满意的解答，并且我已经全部理解。

我同意我的研究医生收集和使用我的医学资料。我同意中国医学科学院肿瘤医院基于科学研究目的查阅本项研究中我的医学资料和研究结果。我同意伦理委员会的成员以及政府管理部门的代表，在遵循保密原则的前提下，按相应权限查阅我的医学资料，我了解查看这些记录是为了确保从这项研究中收集的资料是真实、完整和可靠的。

我明确：我参加本项研究是自愿的，我可以在任何时候退出研究并且不会对我之后的医学治疗或合法权益产生影响。

我已经得到一份已签署的知情同意书的副本。签署这份同意书的时候，我没有放弃任何我的合法权益。

我同意参加本项研究中肿瘤组织/血液等生物标记物检查：

是（请在方格内打勾）☑

否（请在方格内打勾）□

患者签名 袁秀年 日期 2021.1.13

法定代理人签名*________ 日期________ 与患者的关系________

*除非受试者不能阅读（如文盲或盲人）或因其他原因不能自行签名，否则不需要法定代理人的签名

研究者声明

我承诺在临床研究中严格遵守 GCP 原则、遵守国家和中国医学科学院肿瘤医院的相关规定，保护受试者权益，保证研究工作时间和研究数据的真实、完整、可靠。我同意中国医学科学院肿瘤医院基于科学研究目的查阅本项研究的医学资料和研究结果。我同意将研究剩余的生物标本返还中国医学科学院肿瘤医院保存。

我已经向患者充分解释了本项研究的目的、过程，以及参加研究可能存在的风险和益处，患者已经获得了足够的信息来做出参加本项研究的决定。我将为患者或其法定代理人提供一个署名并注明日期的知情同意书副本。

研究者签名 [illegible] 日期 2021.1.13

研究者联系方式 010-87787120
